# High-Resolution Genetic Mapping in the Diversity Outbred Mouse Population Identifies *Apobec1* as a Candidate Gene for Atherosclerosis

**DOI:** 10.1534/g3.114.014704

**Published:** 2014-10-23

**Authors:** Tangi L. Smallwood, Daniel M. Gatti, Pamela Quizon, George M. Weinstock, Kuo-Chen Jung, Liyang Zhao, Kunjie Hua, Daniel Pomp, Brian J. Bennett

**Affiliations:** * Department of Genetics, University of North Carolina Chapel Hill, North Carolina 27599; † The Jackson Laboratory, Bar Harbor, Maine 04609; ‡ Nutrition Research Institute, University of North Carolina Kannapolis, North Carolina 28081; § The Jackson Laboratory for Genomic Medicine, Farmington Connecticut 06030; ** Department of Nutrition, University of North Carolina Chapel Hill, North Carolina 27599

**Keywords:** quantitative trait loci, multiparental models, lipoproteins, atherosclerosis, MPP, multiparental populations, Multiparent Advanced Generation Inter-Cross (MAGIC)

## Abstract

Inbred mice exhibit strain-specific variation in susceptibility to atherosclerosis and dyslipidemia that renders them useful in dissecting the genetic architecture of these complex diseases. Traditional quantitative trait locus (QTL) mapping studies using inbred strains often identify large genomic regions, containing many genes, due to limited recombination and/or sample size. This hampers candidate gene identification and translation of these results into possible risk factors and therapeutic targets. An alternative approach is the use of multiparental outbred lines for genetic mapping, such as the Diversity Outbred (DO) mouse panel, which can be more informative than traditional two-parent crosses and can aid in the identification of causal genes and variants associated with QTL. We fed 292 female DO mice either a high-fat, cholesterol-containing (HFCA) diet, to induce atherosclerosis, or a low-fat, high-protein diet for 18 wk and measured plasma lipid levels before and after diet treatment. We measured markers of atherosclerosis in the mice fed the HFCA diet. The mice were genotyped on a medium-density single-nucleotide polymorphism array and founder haplotypes were reconstructed using a hidden Markov model. The reconstructed haplotypes were then used to perform linkage mapping of atherosclerotic lesion size as well as plasma total cholesterol, triglycerides, insulin, and glucose. Among our highly significant QTL we detected a ~100 kb QTL interval for atherosclerosis on Chromosome 6, as well as a 1.4 Mb QTL interval on Chromosome 9 for triglyceride levels at baseline and a coincident 22.2 Mb QTL interval on Chromosome 9 for total cholesterol after dietary treatment. One candidate gene within the Chromosome 6 peak region associated with atherosclerosis is *Apobec1*, the apolipoprotein B (ApoB) mRNA-editing enzyme, which plays a role in the regulation of ApoB, a critical component of low-density lipoprotein, by editing ApoB mRNA. This study demonstrates the value of the DO population to improve mapping resolution and to aid in the identification of potential therapeutic targets for cardiovascular disease. Using a DO mouse population fed an HFCA diet, we were able to identify an A/J-specific isoform of *Apobec1* that contributes to atherosclerosis.

Identifying novel gene(s) and pathways that regulate susceptibility to atherosclerosis and its underlying risk factors is critically important for biomedical science. Genome-wide association studies (GWAS) have aided in the identification of more than 50 genomic loci associated with atherosclerosis (Holdt and Teupser 2013) and plasma lipid levels (Johansen *et al.* 2011; Swerdlow *et al.* 2012). However, elucidation of the mechanisms underlying novel loci resulting from GWAS studies is challenging, and in most cases such loci only explain a fraction of the genetic variance related to the trait (Hardy and Singleton 2009; Lucas *et al.* 2012; Musameh *et al.* 2014). For example, GWAS have been successfully used to identify more than 40 loci for coronary artery disease, but these loci in aggregate explain less than 10% of the phenotypic variance associated with coronary artery disease (Sayols-Baixeras *et al.* 2014).

Mouse models are well suited for studies of complex traits such as atherosclerosis because environmental conditions and other confounding cofactors can be carefully controlled. This reduces the effects of environmental variation on clinical traits and thus increases the portion of the variance that can be explained by genetics. Recently, a “*Multiparent Advanced Generation Inter-Cross*” (*i.e.*, MAGIC) population was developed from eight inbred strains of mice and is referred to as the Diversity Outbred (DO) mouse population (Churchill *et al.* 2012). The DO mice are mosaics of C57BL6/J, A/J, NOD/ShiLtJ, NZO/HiLtJ, WSB/EiJ, CAST/EiJ, PWK/PhJ, and 129S1/SvImJ, and these mice complement another large endeavor called the Collaborative Cross (Aylor *et al.* 2011). Both the DO and Collaborative Cross populations have tremendous genetic diversity, containing approximately 45 million segregating single-nucleotide polymorphisms (SNPs) (Yang *et al.* 2011). The DO population is maintained by a randomized outbreeding strategy and thus is an ideal resource for high-resolution genetic mapping due to the high allelic diversity and increased number of recombinations represented by this population compared with typical inbred mouse strains (Svenson *et al.* 2012).

In the current study, we use DO mice to study plasma lipids and atherosclerosis. We identify a significant quantitative trail locus (QTL) for atherosclerosis with a submegabase resolution within a locus previously identified as associated with atherosclerosis in mice. Using publically available expression data and expression quantitative trait loci (eQTL) analysis, we identify *Apobec1* as a high-probability candidate gene. We confirm the eQTL for *Apobec1* in the current study using quantitative real-time polymerase chain reaction and quantitate possible coregulation of circulating apolipoprotein B (ApoB) protein levels at this locus.

## Materials and Methods

### Animals and diets

**Figure 1:**
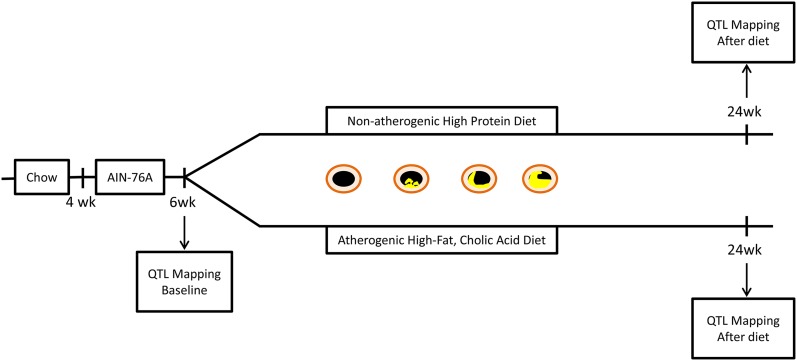
Overall design of the QTL mapping study. Mice obtained from the Jackson Laboratory were fed a chow diet of variable composition before arriving at the University of North Carolina Mouse Facilities at 4 wk of age. Mice were transferred to a controlled synthetic diet from 4 to 6 wk of age (AIN-76A). At 6 wk of age, clinical markers of cardiovascular disease were measured, and baseline QTL mapping was performed. The mice were then transferred to one of two diet groups such that one sibling of each sib pair was randomly assigned to either the high-fat, cholic acid diet group designed to induce atherosclerosis or the high-protein diet group, expected to be nonatherogenic. QTL mapping was then performed in 24-wk-old mice after diet exposure. Quantification of atherosclerotic lesions was performed in the mice at 24 wk of age. QTL, quantitative trait locus.

Female DO mice (n = 292; J:DO, JAX stock number 009376) were obtained from the Jackson Laboratory (Bar Harbor, ME) as 146 full sibling pairs at 4 wk of age and at outbreeding generation 11 (G11) (received September 2012). The mice were group housed (n = 5 mice per cage) with nonirradiated pine bedding and provided with high-efficiency particulate air-filtered air and free access to sterile water in a climate-controlled facility under a 12-hr light/dark cycle. Mice were maintained on a defined synthetic diet, AIN-76A, until 6 wk of age to control for differences due to variable components of standard chow (D10001, Research Diets, New Brunswick, NJ); subsequently, 146 mice were transferred to a synthetic high-fat, cholic acid (HFCA) diet that contained 20% fat, 1.25% cholesterol, and 0.5% cholic acid, to induce atherosclerotic lesions, and146 mice were maintained on a high-protein diet that contained 5% fat and 20.3% protein, which is not atherogenic (D12109C and D12083101, respectively; Research Diets, New Brunswick, NJ). One sibling from each of the 146 sibling pairs was randomly assigned to each one of the diets, Figure 1. The composition of all diets is listed in Supporting Information, Table S1. The source of fat from the diets varied between the baseline diet (corn oil) fed to the mice from 4 to 6 wk of age and the dietary treatment groups (soybean oil plus cocoa butter) fed to the mice from 6 to 24 wk of age. All mice were maintained on their respective diets until 24 wk of age, for a total of 18 wk. All procedures were approved by the IACUC at University of North Carolina (UNC) Chapel Hill (IACUC Protocol Number 11-299).

### Plasma clinical chemistries

To ensure that there were no spurious effects due to the potential variable composition of standard laboratory chow, these mice were maintained on a defined synthetic diet for 2 wk (from 4 to 6 wk of age). At 6 wk of age, the mice were anesthetized using isoflurane and blood was collected after 4 hr of fasting into ethylenediaminetetraacetic acid−containing microtubes from the retro-orbital sinus. This was repeated at 24 wk of age, after 18 wk of dietary treatment. Blood samples were centrifuged for 10 min at 9,000 rcf at 4° and stored at −80°. The plasma levels of total cholesterol, glucose, and triglycerides were quantitated using a Biolis 24i Analyzer (Carolina Liquid Chemistries, Winston-Salem, NC). Insulin was determined using the Alpco Mouse Ultrasensitive Insulin ELISA assay (Alpco Inc, Salem, NH); samples and controls were run in duplicate and optical densities were measured at 450 nm using a microplate reader and analyzed with Gen5 Data Analysis Software (Bio-Tek, Winooski, VT). Data are presented as mean ± SD and significance was determined using a Student’s *t*-test.

### Atherosclerotic lesion size

Hearts, including the proximal aorta, were carefully dissected from 24-wk-old mice, perfused with 1X phosphate-buffered saline, and stored in 10% formalin at 4°. A transverse cut, parallel to the atria, was made to remove the top portion of the hearts, which were then embedded in Optimal Cutting Temperature compound and stored at −80°. Serial sections (10 µm thick) from the aortic sinus were mounted onto slides. Cross sections were arranged eight to a slide and approximately 10−14 total slides were obtained from each mouse heart, for a total of 80−112 cross sections through 400−560 µm of the aortic sinus per animal. Sections were stained with Oil Red O to measure lipid accumulation. To summarize, slides were fixed with formalin for 15 min, rinsed with water then 60% isopropanol, stained for 1 hr in freshly prepared Oil Red O solution, and rinsed with 60% isopropanol then water. Once stained, the slides were imaged using a Zeiss AxioCam MR3 (Zeiss, Munich, Germany), and atherosclerotic lesion area was quantified using Image J software (http://imagej.nih.gov/ij). Data are presented as the average lesion area in µm^2^.

### *Apobec1* mRNA isoform expression

Quantitative polymerase chain reaction was performed in triplicate using a High-Capacity Reverse Transcriptase Kit (Applied Biosystems, Foster City, CA). Following cDNA conversion, 1 µL of sample cDNA, 2 µL of KAPA Sybr Fast qPCR mastermix (KK4610), and 0.5 µM primers were added to each well of a 384-well plate. *Apobec1* primers were custom-designed and ordered from Eurofins MWG Operon (Huntsville, AL). The long transcript was amplified using the following primer set 5′-cagcggtgtgactatccaga-3′ (left primer) and 5′-ttggccaataagcttcgttt-3′ (right primer). This primer set was designed to recognize the known *Apobec1* transcript *Apobec1-001* (ENSMUST00000112586.1). The short transcript was amplified using the following primer set 5′-cccatgagcgttggattc-3′ (left primer) and 5′-tcaaccacgggcagtctt-3′ (right primer). This primer set was designed to recognize the known *Apobec1* transcript *Apobec1-004* (ENSMUST00000143356.1). A serial dilution of pooled samples was used to create a standard curve. Sterile water was used as a negative control.

### Quantification of ApoB in the DO mice

ApoB protein levels were measured using the mouse Apolipoprotein B Sandwich-ELISA method in 96-well format using the ApoB ELISA kit from Elabscience (Wuhan, China) in duplicate following the manufacturer’s instructions. Optical densities were measured at 450 nm using a microplate reader and analyzed with Gen5 Data Analysis Software (Bio-Tek, Winooski, VT).

### Differential expression of genes in peak regions

Data were obtained from The Jackson Laboratory Gene Expression Strain Survey and used for the analysis of differential expression of candidate genes from liver tissue of female C57BL6/J, A/J, NOD/ShiLtJ, NZO/HiLtJ, WSB/EiJ, CAST/EiJ, PWK/PhJ, and 129S1/SvImJ mice (http://cgd.jax.org/gem/strainsurvey26/v1). Mice were maintained for 11 wk on standard chow diet (4% fat content). Genes were identified as differentially expressed between the DO founder strains using analysis of variance and significant between strains differences were calculated using Tukey’s Post Hoc test. We used a Bonferroni correction to determine statistical significance and correct for multiple comparisons.

### Genotyping

DNA was extracted and purified from tail biopsies taken from 6-wk-old mice using QIAGEN DNeasy kit according to the manufacturer’s instructions. Genotyping was performed using the Mega Mouse Universal Genotyping Array (MegaMUGA) by GeneSeek (Neogen, Lansing, MI) (Welsh *et al.* 2012). The MegaMUGA array is built on the Illumina Infinium platform and contains 77,800 SNP markers that are distributed throughout the genome at an average spacing of 33 kb. For the mapping, genomes were reconstructed based on the X and Y allele intensities from the array and founder haplotypes were reconstructed using a hidden Markov model. The reconstructed haplotypes were then used to perform linkage mapping.

### QTL mapping

QTL mapping was performed using the R package DOQTL, version 0.99. To summarize, DOQTL reconstructs the genome in terms of founder haplotypes and performs QTL mapping by regressing the phenotypes on the founder haplotypes with an adjustment for kinship between the mice. Diet was included as an additive covariate for postdiet measurements, except lesion size, which was performed using only the subset of mice fed the HFCA diet (n = 146). Candidate genes were identified by position based on the Wellcome Trust Sanger mouse genomes database, www.sanger.ac.uk, release 1303, based on genome assembly GRCm38 (Yalcin *et al.* 2011). QTL support intervals were defined by the 95% Bayesian credible interval, calculated by normalizing the area under the QTL curve on a given chromosome (Sen and Churchill 2001).

The mapping statistic reported is log of the odds ratio (LOD). The significance thresholds were determined by performing 1000 permutations of genome-wide scans by shuffling phenotypic data in relation to individual genotypes. Significant QTL were determined at a genome-wide *P*-value of <0.05 and suggestive QTL were determined at a *P*-value of <0.63. The latter corresponds to one false positive per genome scan (Lander and Kruglyak 1995).

## Results

### Effects of diet on clinical markers of cardiovascular disease in DO mice

Our studies using the DO mice were designed to examine nutrigenetic or gene × diet interactions and the overall design is outlined in Figure 1. Thus, we used several diets that we outline here. First, to standardize our measurements for this study and our planned future studies, all of the DO mice were placed on a synthetic, chemically-defined diet (AIN-76) upon arrival at the University of North Carolina, Chapel Hill. This diet is henceforth referred to as the baseline diet and was administered for 2 wk. After 2 wk of this defined diet, all 292 DO mice were phenotyped for a variety of clinical traits associated with cardiovascular disease risk. These data were used for QTL mapping. At the end of the 2-wk baseline diet, one mouse from each sibling pair was assigned to one of two diets for subsequent investigation of gene × diet interactions. These diets also were synthetic and chemically defined and included an HFCA diet designed to induce atherosclerosis and a low-fat, high-protein diet not expected to be atherogenic. The DO mice were fed these diets for 18 wk and then phenotyped at 24 wk of age.

There was considerable variation among the mice in the clinical traits measured while the mice were on the baseline diet (Table 1). After we administered the diets, we found there was a robust and statistically significant increase in plasma cholesterol in response to the HFCA diet (199.9 ± 68.6 mg/dL) compared with both the high-protein diet (91.7 ± 25.1 mg/dL) and baseline levels (97.6 ± 31.5 mg/dL) (Figure S1 and Table 1). Conversely, triglyceride levels decreased in response to the HFCA diet (32.3 ± 12.2 mg/dL) compared with both baseline levels (59.3 ± 26.7 mg/dl, *P* < 0.05) and in mice on the high-protein diet (57.7 ± 30.8 mg/dL, *P* < 0.05), (Figure S1 and Table 1).

**Table 1:** Effects of high-protein, high-fat, cholic acid diets on cardiovascular risk factors in the DO mice

	Baseline (AIN-76A)		High Protein		High-Fat, Cholic Acid	
	n	Mean	n	Mean	n	Mean
Cholesterol, mg/dL	277	91.7 ± 25.1	128	97.6 ± 31.5**	136	199.9 ± 68.6*^,^**
Triglycerides, mg/dL	262	59.5 ± 26.5	128	57.7 ± 30.8**	136	32.3 ± 12.1*^,^**
Glucose, mg/dL	257	155.2 ± 43.8	130	190.5 ± 49.9	137	177.9 ± 45.1
Insulin, ng/mL	235	0.8 ± 0.4	129	1.7 ± 1.1*	133	1.4 ± 0.7*

The values shown are means ± SD. Plasma clinical chemistries were measured at baseline (in 6-wk-old mice), after 2 wk on the synthetic-defined diet AIN-76A and with 4 hr fasting. Mice were transferred to one of two diets, either a high-fat, cholic acid diet or a low-fat, high protein diet, and maintained on these diets for 18 wk. Plasma clinical chemistries were measured again (in 24-wk-old mice) after diet treatment and with 4 hr fasting. Significant differences between the baseline diet measures and either diet treatment group (*, where *P* < 0.05) or significant differences between the two different diets after 18 weeks of diet treatment (**, where *P* < 0.05) are indicated.

### Identifying QTL for clinical markers of cardiovascular disease in DO mice at baseline

**Figure 2:**
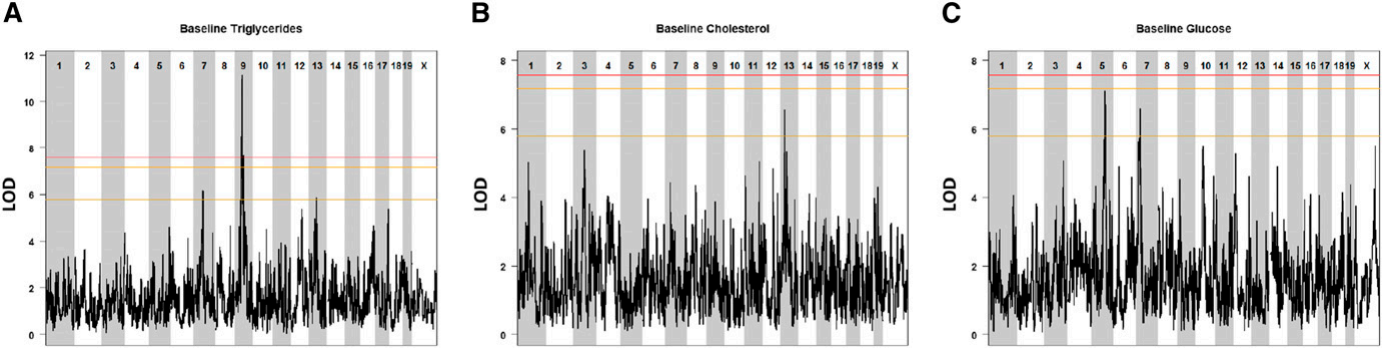
QTL mapping of clinical markers of cardiovascular disease in 6-wk-old DO mice at baseline. Genome-wide QTL scans for loci affecting plasma levels of triglycerides (A), total cholesterol (B), and glucose (C) in the DO population at baseline. Chromosomes 1 through X are represented numerically on the x-axis, and the y-axis represents the LOD score. The relative width of the space allotted for each chromosome reflects the relative length of each chromosome. Mice were maintained on a synthetic diet for 2 wk and then phenotyped for plasma clinical chemistries at 6 wk of age. Colored lines show permutation-derived significance thresholds (N = 1000) at *P* = 0.05 (LOD = 7.57, shown in red), *P* = 0.10 (LOD = 7.17, shown in orange), and *P* = 0.63 (LOD = 5.79, shown in yellow). QTL, quantitative trait locus; DO, Diversity Outbred; LOD, log of the odds ratio.

**Figure 3:**
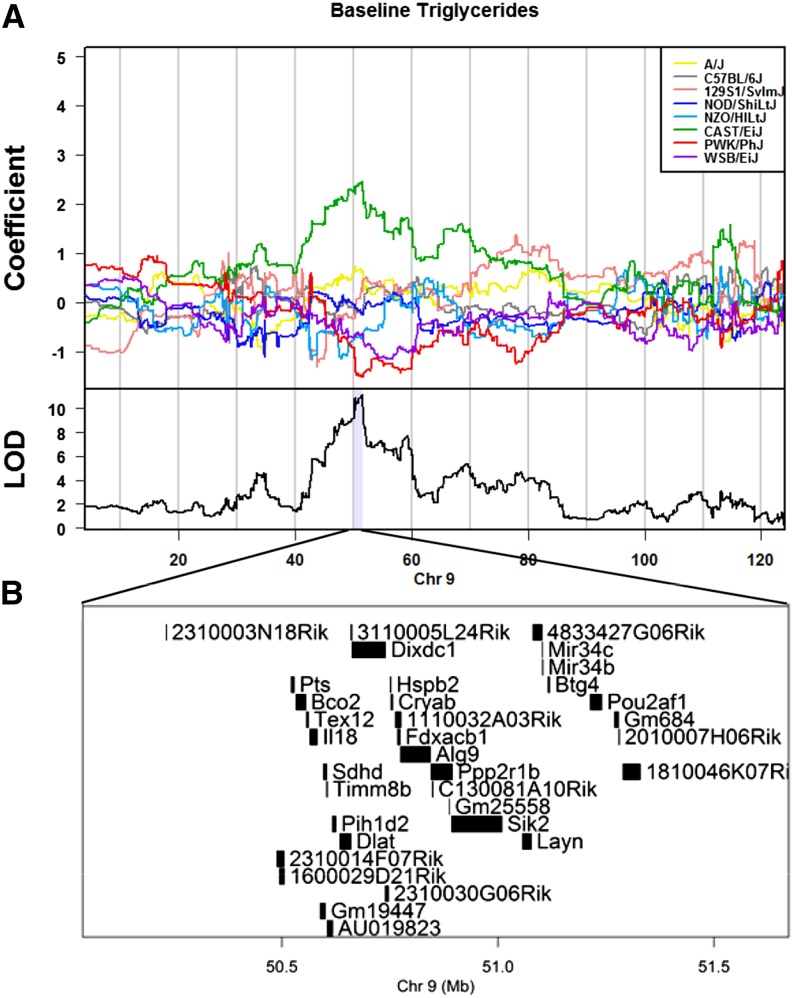
High-resolution mapping of significant hits on Chromosome 9 for plasma triglyceride levels. The eight coefficients of the QTL model show the effect of each founder haplotype on the phenotype. The model coefficients for the mapping of baseline triglycerides are plotted for each founder allele at each marker along Chromosome 9 and shading identifies the 95% Bayesian estimated interval around the peak (A). There are 34 potential candidate genes within the Chromosome 9 locus associated with plasma triglycerides at baseline (B). QTL, quantitative trait locus.

We performed QTL mapping for several clinical markers of cardiovascular disease in 6-wk-old mice fed the baseline (AIN-76) diet. Significant QTL were determined at a genome-wide *P*-value of <0.05 and the QTL support interval was defined using the 95% Bayesian credible interval (Table 2). We identified a highly significant QTL for triglycerides on Chromosome 9 with a peak SNP located at 51.4 Mb (LOD = 11.3; n = 262 mice) (Figure 2A). DO mice carrying the CAST/EiJ allele at the Chromosome 9 QTL have greater triglyceride levels (Figure 3A). There are 34 candidate genes within the QTL interval on Chromosome 9 (Figure 3B). We identified suggestive QTL for total cholesterol at baseline on Chromosome 13 with a peak SNP located at 30.4 Mb (LOD = 6.5; n= 277 mice) (Figure 2B) and for glucose at baseline on Chromosomes 5 and 7 with peak SNPs located at 92.6 Mb on Chromosome 5 (LOD = 7.0; n = 257 mice) and 27.2 Mb on Chromosome 7 (LOD = 6.5; n = 257 mice) (Figure 2C).

**Table 2:** QTL for cardiovascular risk factors in the DO mice at baseline and after dietary treatment

Phenotype	Chr	Peak LOD	Position (Interval, Mb)	Significant (*P* < 0.05)	Previously Known QTL (Interval, Mb)	KnownGene Candidates	Refs.
Baseline							
Triglycerides	9	11.3	51.4 (50.2−51.6)	Yes	*Trigq1* (40−70)	*ApoA5*	Suto and Sekikawa 2003
Total cholesterol	13	6.5	30.4 (28.7−43.8)	No	*Hmgcs1* (20−100)	*Hmgcr*	Welch 1996
Glucose	5	7.0	92.6 (86.3−99.0)	No	*Bglu13* (84−110)	*Hnf1a*, *Pdx1*	Zhang *et al.* 2012
Insulin	19	5.4	58.6 (22.6−60.8)	No	*Tanidd1* (36−61)	*Not Determined*	Kim 2001
After diet							
Triglycerides	12	5.8	97.6 (49.6−102.3)	No	*Tglq2* (55−89)	*ApoB*	Srivastava 2006
Total cholesterol	9	7.5	48.3 (47.8−70.0)	Yes	*Cq4* (15-50 Mb), Cq5 (32−66)	*ApoA4*	Suto and Sekikawa 2003
Glucose	12	5.7	70.9 (26.7−75.1)	No	*Bglu15* (10−42)	*Adam17*	Zhang *et al.* 2012
Insulin	13	7.0	8.7 (5.7−10.4)	No	NA	NA	NA

Clinical markers of cardiovascular disease were measured in study animals after 4 hr of fasting at both 6 and 24 wk of age. Baseline measures are from 6-wk-old fasted mice fed a synthetic diet. Measures at 24 wk of age, after diet, include mice on both the low-fat, high protein diet and the high-fat, cholic acid diet; diet was used as a covariate in the analysis. The statistic reported is log of the odds ratio (LOD). QTL, quantitative trait loci; DO, Diversity Outbred; NA, not available.

### Identifying QTL for clinical markers of cardiovascular disease in the DO after dietary treatment

**Figure 4:**
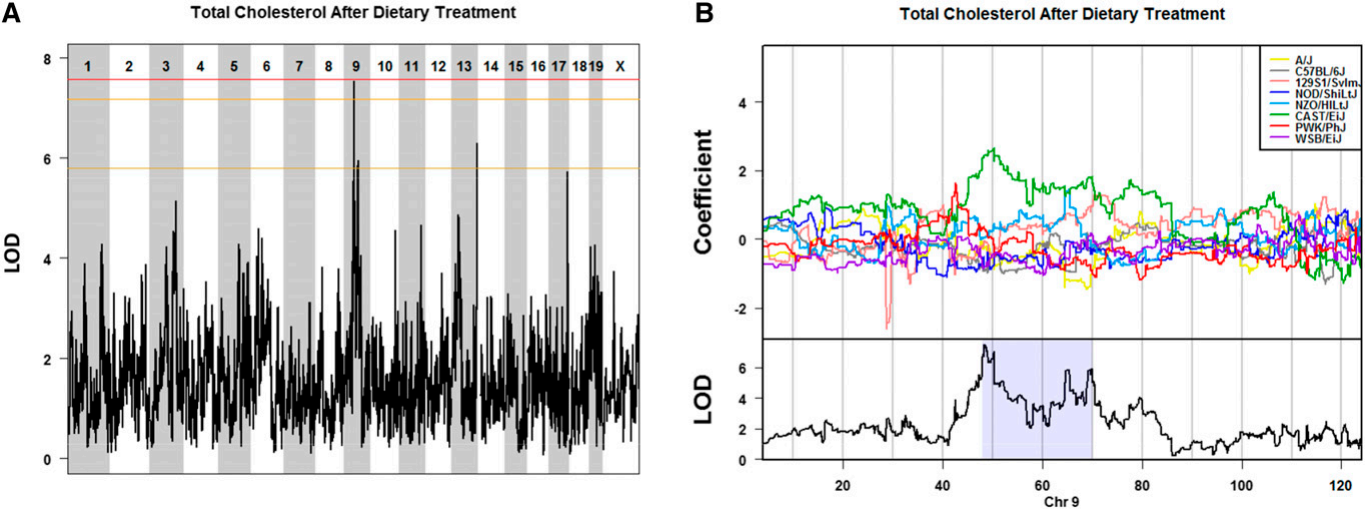
Effect of diet on total cholesterol in the DO mice. Genome-wide QTL scan for loci affecting plasma levels of total cholesterol after 18 wk of dietary treatment (A). Chromosomes 1 through X are represented numerically on the x-axis, and the y-axis represents the LOD score. The relative width of the space allotted for each chromosome reflects the relative length of each chromosome. Plasma was taken from 24-wk-old mice after 18 wk of dietary treatment. Colored lines show permutation-derived significance thresholds (N = 1000) at *P* = 0.05 (LOD = 7.57, shown in red), *P* = 0.10 (LOD = 7.17, shown in orange), and *P* = 0.63 (LOD = 5.79, shown in yellow). The eight coefficients of the QTL model show the effect of each founder haplotype on the phenotype. Shading identifies the 95% Bayesian credible interval around the peak (B). DO, Diversity Outbred; QTL, quantitative trait locus; LOD, log of the odds ratio.

To assess the effects of diet in the DO, we determined the genetic architecture of several markers of cardiovascular disease by quantifying these phenotypes at 24 wk of age after dietary treatment. Because diet had significant effects on the phenotypes, it was included in the mapping model as an additive covariate. We observed a significant QTL for plasma cholesterol on Chromosome 9 with a peak LOD score of 7.54 at 48.3 Mb (47.84−70.04 Mb) (Figure 4A and Table 2). The CAST/EiJ founder haplotype was associated with high levels of cholesterol based on the founder allele contribution in the region (Figure 4B). This is a relatively large QTL interval of 22.2 Mb based on the Bayesian credible interval (*P* = 0.95) and the entire interval contains 391 known and predicted genes. The peak SNP is within 3 Mb of the peak SNP identified by mapping of baseline triglyceride levels in this population of mice, suggesting that this genes at this locus are critical in the overall regulation of lipid metabolism.

### Differential expression of candidate genes in DO founder strain mice

To prioritize candidate genes within the QTL intervals we identified, we analyzed hepatic gene expression among the founder strains for the 34 genes in the region from a publically available dataset (http://cgd.jax.org/gem/strainsurvey26). We first identified genes whose expression varies across the progenitor strains using 1-way analysis of variance analysis and using a Bonferroni correction based on the number of probes tested (0.05/68 probes representing the 34 genes in the locus). Based on this analysis, we identified 10 probes representing 9 genes that are differentially expressed: *1110032A03Rik*, *1810046K07Rik*, *Alg9*, *Bco2*, *Cryab*, *Gm684*, *Ppp2r1b*, *Pts*, and *Sik2* (Figure S2, A−I).

The DOQTL package identifies the contribution of the founder alleles to the QTL. For the Chromosome 9 triglyceride QTL, we observed that allelic contribution from the CAST/EiJ founder at this locus is associated with greater triglyceride levels, whereas comparatively low triglyceride levels are associated with allelic contribution from the other seven founder strains (Figure 3A). Thus, we next performed pair-wise comparisons using Tukey’s HSD for the nine differentially expressed genes to identify genes that are differentially expressed in CAST/EiJ compared with the other founder strains and thus match the overall allelic effects at the QTL. Of the nine genes differentially expressed, *Bco2* and *Ppp2r1b* most closely match the allele effects of the QTL and are differentially expressed between CAST/EiJ and the other progenitor strains, Figure S2, A−B. We note that the locus we identified is adjacent to a region harboring genes known to influence plasma lipid levels, including *Apoa1*, *Apoa4*, *Apoa5*, and *Nnmt*. Both *Apoa4* and *Nnmt* are differentially expressed among the strains, but only *Nnmt* is differentially expressed in CAST/EiJ mice (Figure S3).

### Identification of *Apobec1* as a candidate for regulating lesion size in DO mice

Atherosclerosis is a complex trait and studies using mice have identified numerous QTL for this trait (Wang *et al.* 2005); but this trait has not yet been evaluated in the DO mice. Based on previous studies, a HFCA diet can induce formation of atherosclerotic lesions (Paigen *et al.* 1987). Indeed, we found that none of the mice fed the high-protein diet exhibited any lesions, and our subsequent analyses focus only on the subset of DO mice that were fed the HFCA diet.

**Figure 5:**
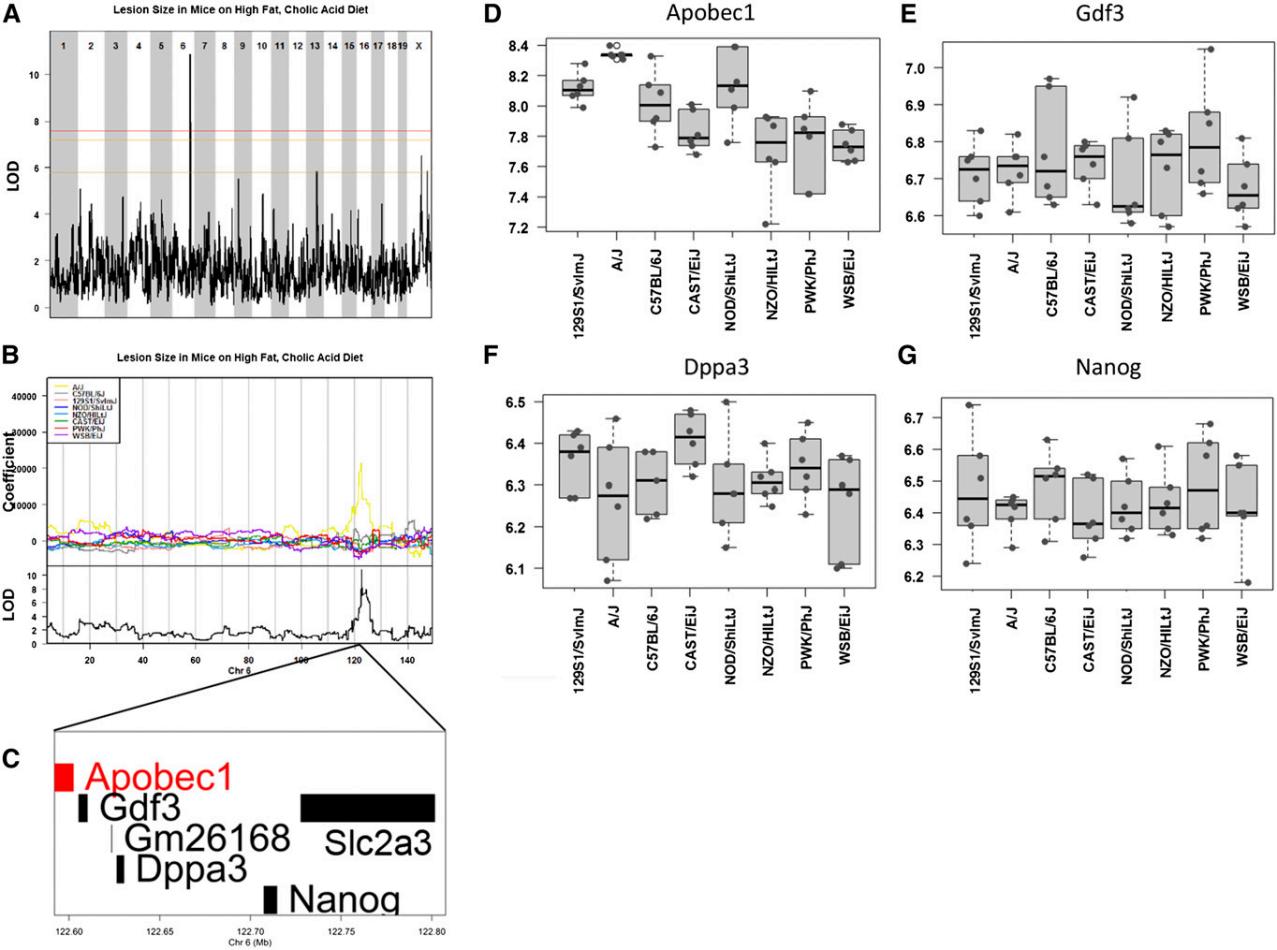
QTL mapping of atherosclerosis in the DO mice. Genome-wide QTL scan for loci affecting atherosclerotic lesion size in mice fed a high-fat, cholic acid diet (A). Chromosomes 1 through X are represented numerically on the x-axis, and the y-axis represents the LOD score. The relative width of the space allotted for each chromosome reflects the relative length of each chromosome. Hearts were harvested from 146 mice after 18 wk on a high-fat, cholic acid diet. Colored lines show permutation-derived significance thresholds (N = 1000) at *P* = 0.05 (LOD = 7.57, shown in red), *P* = 0.10 (LOD = 7.17, shown in orange), and *P* = 0.63 (LOD = 5.79, shown in yellow). The eight coefficients of the QTL model show the effect of each founder haplotype on the phenotype. A/J founder alleles are associated with larger lesion size in the DO mice (B). There are six candidate genes within the 100,000-kb QTL interval on Chromosome 6: *Apobec1*, *Gdf3*, *Dppa3*, *Nanog*, *Slc2a3*, and the predicted gene *Gm26168* (C). Gene expression data were obtained from livers from female C57BL6/J, A/J, NOD/ShiLtJ, NZO/HiLtJ, WSB/EiJ, CAST/EiJ, PWK/PhJ, and 129S1/SvImJ mice (http://cgd.jax.org/gem/strainsurvey26). Of the six candidate genes in the QTL interval on Chromosome 6, four were assayed by microarray for hepatic gene expression: *Apobec1* (D), *Gdf3* (E), *Dppa3* (F), and *Nanog* (G). *Apobec1* is the only candidate in this region that matches our allele effects such that A/J mice specifically express higher levels of *Apobec1*. QTL, quantitative trait locus; DO, Diversity Outbred; LOD, log of the odds ratio.

We found that 76% of the DO mice were susceptible to atherosclerosis with lesions induced by the HFCA diet, with a range in lesion size from 38 to 33,200 µm^2^. Based on this highly variable phenotype, we were able to identify a highly significant QTL on Chromosome 6 (LOD = 10.7; 122.6−122.7 Mb) (Figure 5A). Although our Chromosome 6 peak SNP, UNC11996440, is within the 95% confidence interval of a previously reported QTL, *Ath37*, the interval is refined to sub-Mb resolution using the DO. The 95% confidence interval reported for this previously defined QTL encompasses an 11.8-Mb interval, whereas our Chromosome 6 atherosclerosis peak maps to a refined 100,000-kb region containing six genes (Figure 5C). One candidate gene within the Chromosome 6 peak region is *Apobec1*, the apolipoprotein B mRNA-editing enzyme, which plays a role in the regulation of ApoB, a critical component of LDL, by editing *ApoB* mRNA to produce the short ApoB48 isoform. Misregulation of *Apobec1* results in altered *ApoB* isoform editing. For example, *Apobec1*^−/−^ mice have greater levels of ApoB100 compared with the edited isoform, ApoB48 (Nakamuta *et al.* 1996) and transgenic rescue of *Apobec1*in *Apobec1*^−/−^ animals has been shown to directly alter chylomicron production (Blanc *et al.* 2012). *Apobec1* is an attractive candidate gene for influencing diet-induced lesion size based on the known function of *Apobec1* in lipid homeostasis and a causal allele for *Apobec1* has not been previously identified as associated with atherosclerosis. Additionally, we found that total cholesterol levels after dietary treatment were significantly correlated with both the short and long isoforms of *Apobec1* (r = 0.6 and r = 0.57, respectively), but not ApoB levels in these mice (r = 0.06).

### A/J specifically expresses a long isoform of *Apobec1*, which is induced in response to a HFCA diet

We found that DO mice containing the A/J allele at the Chr 6 QTL had larger aortic lesions (Figure 5B). Among genes in the Chr 6 QTL interval, only *Apobec1* has significant differential hepatic expression in the A/J strain, which expresses greater levels of *Apobec1* (*P* < 0.0125) (Figure 5, D−G). Interestingly, strain-specific differences in isoform expression of *Apobec1* were recently identified between A/J and C57BL/6J mice, where C57BL/6J mice express a truncated Apobec1 protein (Hassan *et al.* 2013). More importantly, using AXB and BXA recombinant inbred strains they demonstrate genetic regulation of mRNA editing by *Apobec1*, such that animals expressing the A/J-specific isoform of *Apobec1* exhibit greater editing efficiency of Apobec1 targets compared with C57BL/6J animals.

**Figure 6:**
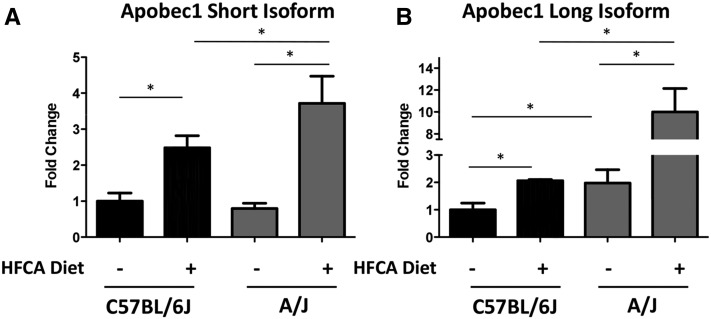
A/J preferentially expresses the long isoform of *Apobec1* in response to a high-fat, cholic acid diet. *Apobec1* expression levels of the short (A) and long (B) isoforms from RNA from liver tissue from A/J and C57BL/6J founder strain mice. A/J mice on a high-fat, cholic acid diet exhibit increased expression of both the long and short transcripts of *Apobec1* in a diet-dependent manner, *P > 0.05. *Apobec1* levels for each sample were normalized relative to RPS20. Fold changes are reported as the relative expression in A/J *vs.* C57BL/6J samples. Data are presented as mean ± SD, and significance was determined using a Student’s *t*-test.

We quantitated the expression of *Apobec1* in liver tissue from A/J and C57BL/6J mice fed either the HFCA diet or the control synthetic diet and found that on the synthetic diet A/J expresses more of the long isoform of *Apobec1* compared with C57BL/6J (Figure 6B), whereas A/J and C57BL/6J express similar levels of the short isoform of *Apobec1* while on a synthetic diet (Figure 6A). In response to the HFCA diet, expression of both isoforms of *Apobec1* is significantly increased in both A/J and C57BL/6J mice compared with *Apobec1* levels in the mice on the synthetic diet (*P* > 0.05). The short isoform of *Apobec1* is induced 2.5-fold in C57BL/6J animals and 3.5-fold in A/J animals in response to the HFCA diet. The long *Apobec1* isoform is induced 2.5-fold in C57BL/6J animals and fourfold in A/J animals.

### *cis*-eQTL for *Apobec1* exhibit isoform-specific allele effects patterns in the DO mice

Based on the differences in expression of *Apobec1* between A/J and C57BL/6J mice, we hypothesized that the expression of *Apobec1* may be genetically regulated. Thus, we surveyed two publically available databases for expression QTL data to determine whether a natural variant near the *Apobec1* gene affects its expression. Specifically, we queried a panel of inbred strains of mice called the hybrid mouse diversity panel and The Jackson Laboratory’s Diversity Outbred eQTL viewer, data located at http://systems.genetics.ucla.edu/data/hmdp and http://cgd.jax.org/apps/eqtlviewer-beta/, respectively (Ghazalpour *et al.* 2012). *Apobec1* expression varied significantly among the hybrid mouse diversity panel strains and the major locus regulating expression mapped directly over the *Apobec1* gene, at 122 Mb on Chromosome 6 (data not shown). Similarly, the Jackson Laboratory’s DO eQTL viewer identifies a *cis*-eQTL associated with *Apobec1* expression (Figure S4A). Similar to our results, these data demonstrate that the A/J allele is associated with greater expression of *Apobec1* (Figure S4B).

**Figure 7:**
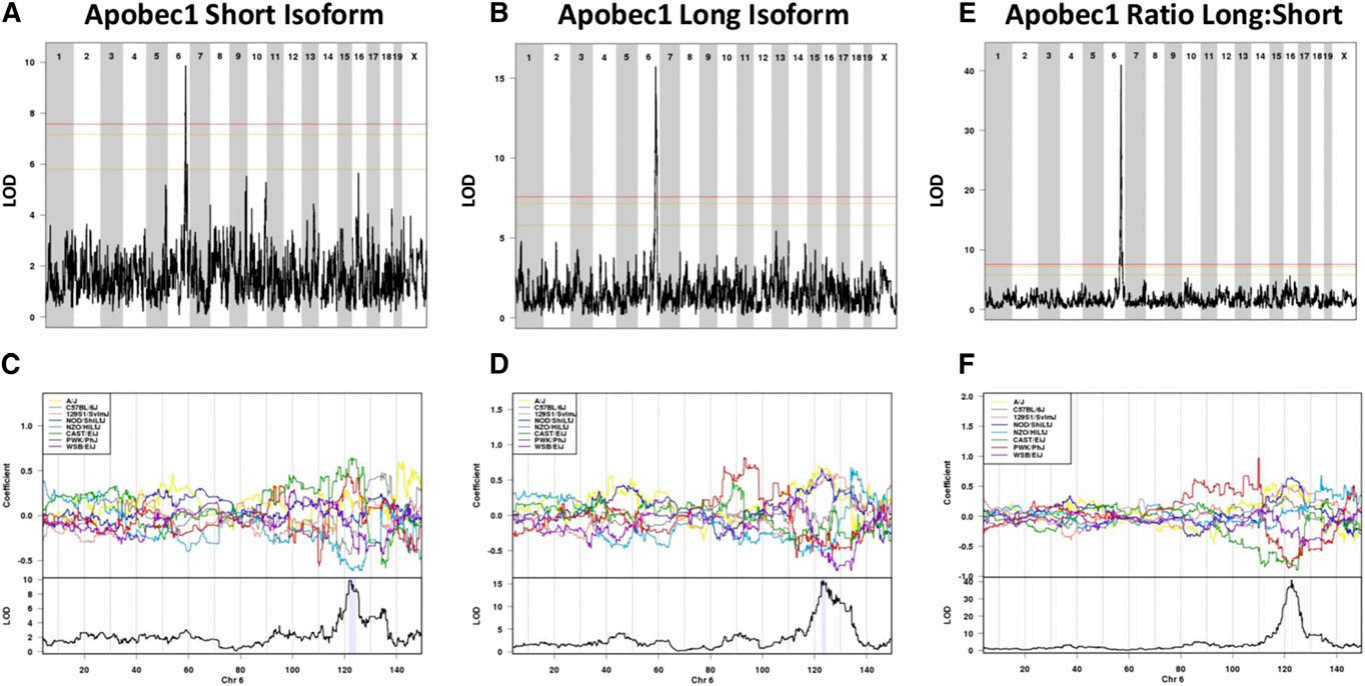
*Cis*-eQTL for hepatic *Apobec1* short and long isoforms in the DO mice. Genome-wide QTL scan for eQTL regulating expression of the short (A) and long (B) *Apobec1* isoforms in the DO mice. Chromosomes 1 through X are represented numerically on the x-axis, and the y-axis represents the LOD score. The relative width of the space allotted for each chromosome reflects the relative length of each chromosome. Colored lines show permutation-derived significance thresholds (N = 1000) at *P* = 0.05 (LOD = 7.57, shown in red), *P* = 0.10 (LOD = 7.17, shown in orange), and *P* = 0.63 (LOD = 5.79, shown in yellow). The eight coefficients of the QTL model show the effects on the phenotype contributed by each founder haplotype on Chromosome 6 for mapping of the short (C) and long (D) isoforms of *Apobec1*. Shading identifies the 95% Bayesian credible interval around the peak. eQTL, expression quantitative trait loci; DO, Diversity Outbred; LOD, log of the odds ratio.

Considering our observations of the effect of genetic background on *Apobec1* expression in liver tissue and recent observations that mouse genetic background regulates isoform usage in macrophages, we next asked whether there was a difference in the genetic regulation of each of these isoforms among the DO founder strains. We performed QTL mapping of mRNA expression levels of either the short or long isoform of *Apobec1* from RNA isolated from liver tissue from the present DO mouse study population. We identified highly significant *cis*-eQTL for both isoforms on Chromosome 6 with the peak SNP located at 121.8 Mb with a maximum LOD score of 9.9 associated with expression of the short isoform (*P* < 0.05, n = 252 mice) and at 123.4 Mb with a maximum LOD score of 15.7 associated with expression of the long isoform (*P* < 0.05, n = 251 mice) (Figure 7, A and B), respectively. When we estimate the effect of each founder at each marker along Chromosome 6, we see that CAST/EiJ alleles are associated with greater expression of the short isoform (Figure 7C), whereas A/J alleles are associated with greater expression of the long isoform (Figure 7D). We also mapped the ratio of *Apobec1* isoforms and observe a highly significant *cis*-eQTL with a max LOD score of 41.0 at 122.6 Mb (*P* < 0.05, n = 251 mice) (Figure 7, E and F).

### *Apobec1* and ApoB levels are dependent on the genotype of UNC11996440, the Chromosome 6 peak SNP associated with atherosclerosis

**Figure 8:**
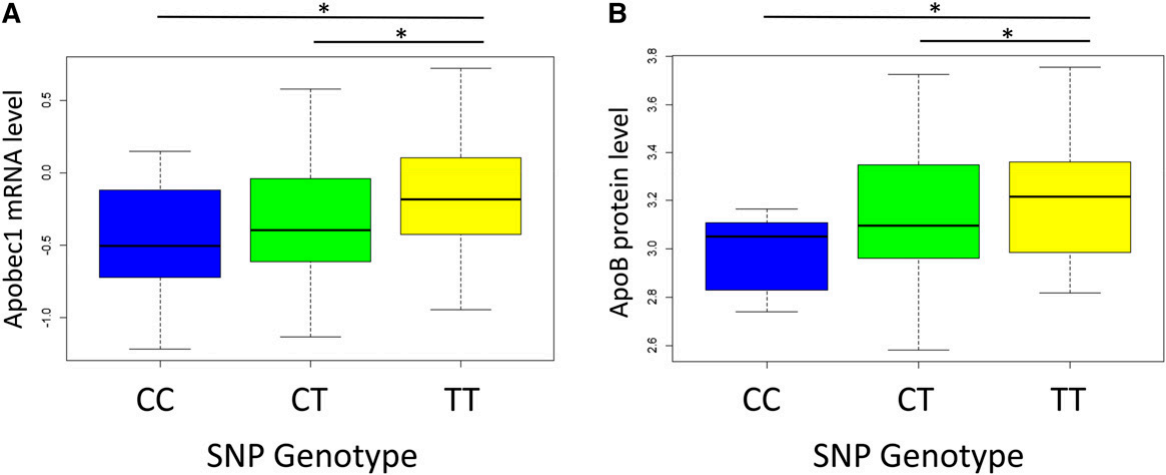
Apobec1 and ApoB levels are dependent on the genotype of UNC11996440, the Chromosome 6 peak SNP associated with atherosclerosis. Genotyping was performed using the Mega Mouse Universal Genotyping Array (MegaMUGA). *Apobec1* mRNA levels were measured by quantitative polymerase chain reaction from liver tissue from the DO mice. Expression levels of the *Apobec1* long isoform differed significantly between genotype classes (*P* > 0.001) (A). ApoB protein levels were measured in a subset of the mice phenotyped for atherosclerosis (N= 80 mice) using a mouse Apolipoprotein B Sandwich-ELISA method. ApoB expression levels differed significantly between the genotype classes (*P* > 0.001) (B).

Based on the association of the A/J founder with atherosclerotic lesion size, we hypothesized that a causal allele located in the *Apobec1* sequence would be private to A/J. However, there are no documented SNPs that are private to A/J within the refined peak region of 122.6−122.7 Mb on Chromosome 6. The peak SNP for this locus, UNC11996440, is located distal to the *Apobec1* gene on Chromosome 6 at 122.67 Mb, whereas *Apobec1* lies between 122.5 and 122.6 Mb. In addition to the *cis*-eQTL data for *Apobec1* expression, this finding suggests that a distant SNP affecting *Apobec1* expression may be responsible for the association between the A/J haplotype and atherosclerotic lesion size. However, if the association of the A/J haplotype with increased lesion size in the DO mice is mechanistically related to *Apobec1* expression, then we would expect *Apobec1* expression levels to differ based on the genotype of UNC11996440. We identified T as the major allele (frequency = 0.69) and C as the minor allele (frequency = 0.31) at the tagging SNP UNC11996440. The genotype at this SNP significantly affects *Apobec1* mRNA expression levels (*P* > 0.001) (Figure 8A). We next hypothesized that the association at this locus may be mechanistically related to the role of Apobec1 in editing its primary target, ApoB, as elevated plasma ApoB is a known risk factor for atherosclerosis. Therefore, we quantified plasma ApoB levels in duplicate using two mouse-specific ApoB Sandwich-ELISA plates (N = 80 mice). Plasma samples from only the HFCA diet-fed mice were used and samples were chosen to represent a range of atherosclerotic lesion sizes. As shown in Figure 8B, ApoB levels are significantly different between the genotype groups at the tagging SNP UNC11996440 (*P* > 0.001). Therefore, in addition to identifying the A/J-specific isoform of *Apobec1* as associated with atherosclerotic lesion size in the DO mice, these data suggest that this association may be attributable to the role of *Apobec1* in editing ApoB.

## Discussion

The DO was designed to be a high-resolution genetic mapping panel. The use of DO mice for mapping offers a number of advantages over classical approaches to mouse genetics, including high mapping resolution, increased heterozygosity, and uniformly distributed genetic variation across the genome (Svenson *et al.* 2012). Using the newly developed DO mouse resource, we were able to refine the positions and identify new candidate genes for previously mapped QTL for atherosclerosis, a highly complex phenotype associated with human disease. We further interrogate this QTL by using publically available data, by quantitating mRNA levels for *Apobec1*, and by investigating the protein target of Apobec1 to identify a potential mechanism for this QTL. We discuss each of these in detail.

### Atherosclerosis in the DO

Atherosclerotic lesion development is the most common cause of cardiovascular disease. Here, we characterize for the first time the development of diet-induced atherosclerotic lesions in the newly developed DO mouse population. In the present study, 292 mice on either a HFCA diet designed to induce atherosclerotic lesions or a nonatherogenic, low-fat, high-protein diet were phenotyped for atherosclerosis. We found that none of the mice fed the high-protein diet exhibited any lesions. We found that 76% of the DO mice were susceptible to lesions induced by the HFCA diet, with a range in lesion size from 38 to 33,200 µm^2^. We detected one locus on Chromosome 6 for atherosclerosis that maps within the 95% confidence interval of a previously reported QTL, *Ath37* (Ghazalpour *et al.* 2006), which was identified in studies using sub-congenic mice between C57BL/6J and CAST/EiJ. The locus in the current study contains *Apobec1*, which was originally described as the enzyme responsible for deamination of a cytosine in mature ApoB mRNA, resulting in a premature stop codon and production of the truncated protein ApoB48. Lipoproteins containing ApoB48 are more efficiently cleared from the circulation as demonstrated by adenoviral overexpression studies that reduce plasma lipid levels (Teng *et al.* 1997); in contrast, *Apobec1^−/−^* mice synthesize only ApoB100 and have increased atherosclerosis when crossed to *Ldlr^−/−^* mice (Powell-Braxton *et al.* 1998).

Closer examination of the locus we identified here indicates that A/J mice contain a susceptible allele, which is unexpected as A/J has classically been defined as an atherosclerosis-resistant mouse strain (Paigen *et al.* 1985). However, it is possible that if *Apobec1* editing is affected by an allele carried by the A/J strain, this effect could be masked by transgressive variation or epistasis in inbred A/J animals. When we queried the Sanger SNP database for alleles private to A/J within 10 kb of the *Apobec1* gene sequence (122,567,890−122,612,426 Mb), we found that there are no documented SNPs that are private to A/J. However, the association of distant SNPs influencing gene expression of genes as far as 300 kb away is not uncommon (Guenther *et al.* 2014). Although our data suggest that a distant SNP affecting *Apobec1* expression may be responsible for the association between the A/J haplotype and atherosclerotic lesion size, we have not yet ruled out an association between lesion size and the other five genes in the locus, nor have we ruled out nearby genes with A/J-specific alleles. Additionally, in our study we did not find lesion size to be correlated with *Apobec1* expression (r = −0.02) although total cholesterol was correlated with this phenotype (r = 0.60).

Recently, Hassan *et al.* (2013) have reported that genetic differences between C57BL/6J and A/J at the *Apobec1* gene affect global RNA editing patterns between these strains. Indeed, this study identified an A/J-specific isoform of *Apobec1* in murine macrophages that increased the editing efficiency of this enzyme for ApoB as well as other *Apobec1* targets (Rosenberg *et al.* 2011; Hassan *et al.* 2013). Thus, differences in *Apobec1* structure or expression could affect editing or perhaps overall levels of ApoB or another of Apobec1’s target genes. Our initial studies, in a subset of the DO mice used in this study, indicate that total ApoB levels may be influenced by this locus.

### Mapping clinical markers of cardiovascular disease in the DO

Measures of cholesterol, triglycerides, and glucose are commonly used markers of cardiovascular disease. Therefore, we were interested in investigating the genetic architecture of these traits in the DO mice. We identified highly a significant QTL for triglycerides in mice at baseline. The significant QTL on Chromosome 9 for baseline triglycerides is 1.4 Mb in size and is coincident with a ∼30 Mb QTL previously identified as associated with triglyceride levels in C57BL/6J x KK-Ay/a F2 mice, *Trigq1*. *Trigq1* maps to 61 Mb on Chromosome 9 with a peak LOD score of 4.2 at the marker D9Mit163 (Suto and Sekikawa 2003). The large *Trigq1* locus includes multiple gene candidates known to regulate lipid levels such as the *ApoA5-ApoA4-ApoA3-ApoA1* gene cluster and *Lipc*. Although the *ApoA5-ApoA4-ApoA3-ApoA1* gene cluster is located at 46.2 Mb, just proximal to the QTL interval we identified here and *Lipc* is located 20 Mb distal to our peak region, these genes would seem to be excluded based on our results. However, at least one of these genes, *ApoA5*, has been well characterized as associated with triglyceride levels, (Charlton-Menys and Durrington 2005). In humans, a mutation in *ApoA5*, also called the Delhi gene, is linked to extremely elevated triglyceride levels and increased risk of cardiovascular disease (Sinha *et al.* 2010; Ramakrishnan *et al.* 2011). Hepatic lipase, encoded by the *Lipc* gene, hydrolyzes triglycerides and phospholipids in lipoprotein particles and is therefore also likely to be functionally associated with heart disease. We used publically available gene expression data to prioritize genes whose expression may be genetically regulated. These analyses are not able to identify candidate genes that have a functional variant that affects protein function, protein stability, or binding. Further study to confirm that one of the genes in the triglyceride QTL identified in this study is a causal gene remains to be performed.

This region of Chromosome 9 clearly contains multiple genes that are important for the regulation of cardiovascular disease risk factors. We also identified the same region as associated with total cholesterol after dietary treatment in our DO mouse population. Additionally, the human syntenic region of the Chromosome 9 QTL we identified as associated with both baseline triglycerides and total cholesterol after dietary treatment has been identified in several human GWAS studies as associated with multiple atherosclerosis risk factors across multiple human populations (Hu *et al.* 2013; Weissglas-Volkov *et al.* 2013; Zhou *et al.* 2013). When one QTL contains more than one causal variant, the Bayesian interval calculation may fail to differentiate genetic signals from the closely linked genes. Several studies have suggested the use of 2-LOD support intervals for generating conservative estimates of genes for candidate testing. Indeed, in our study the 95% Bayesian credible intervals represent approximately 1-LOD support intervals for baseline triglycerides and for total cholesterol after diet treatment and if we broaden these intervals to 2-LOD support intervals, the *ApoA5-ApoA4-ApoA3-ApoA1* gene cluster and *Lipc* gene falls within the more conservative interval estimate.

We identified a suggestive QTL for total cholesterol on Chromosome 13 with a peak SNP located at 30.4 Mb (LOD = 6.5; n = 277 mice) and for glucose on Chromosome 5 with a peak SNP located at 92.6 Mb (LOD = 7.0; n= 257 mice). These suggestive QTL co-localize with previously reported QTL. The Chromosome 13 locus associated with baseline cholesterol levels is just proximal to *Lipq2*, which was identified in a backcross between MOLF/EiJ and C57BL/6J mice on with an *Ldlr*^−/−^ mutation (Welch *et al.* 2001). The suggestive QTL associated with blood glucose on Chromosome 5 is less than 2 Mb from the peak SNP reported for the QTL, *Bglu13*, which was identified in an F2 intercross cross of mutant C3H/HeJ and C57BL/6J carrying the ApoE^−/−^ mutation (Zhang *et al.* 2012).

In summary, we demonstrate here the use of the DO mice for high-resolution mapping of traits related to atherosclerosis. We identify several candidate genes for triglycerides in mice on a synthetically defined diet. Perhaps the most interesting result is the identification of an atherosclerosis QTL on Chromosome 6 that is 100 kb in size. This locus contains the candidate gene *Apobec1* which is known to alter circulating lipoprotein composition, but also has widespread RNA editing capabilities, perhaps indicating an additional mechanism by which susceptibility to atherosclerosis is regulated.

## Supplementary Material

Supporting Information
