# Supplementary material for: High-Resolution Genetic Mapping in the Diversity Outbred Mouse Population Identifies Apobec1 as a Candidate Gene for Atherosclerosis
Source: G3 (Bethesda). 2014 Oct 23;4(12):2353–63. doi: 10.1534/g3.114.014704 (PMC4267931; doi:10.1534/g3.114.014704)
Supplement: Supporting Information [file supp_4_12_2353__index.html]

High-Resolution Genetic Mapping in the Diversity Outbred Mouse Population Identifies Apobec1 as a Candidate Gene for Atherosclerosis — Supporting Information 

# High-Resolution Genetic Mapping in the Diversity Outbred Mouse Population Identifies *Apobec1* as a Candidate Gene for Atherosclerosis

## Supporting Information for Smallwood *et al.*, 2014

**Files in this Data Supplement:**

- Supporting Information - Figures S1-S4 and Table S1 (PDF, 592 KB)
- Figure S1 - Effects of Diet on Cardiovascular Risk Factors in Diversity Outbred Mice. (PDF, 318 KB)
- Figure S2 - Liver expression of candidate genes in the Chromosome 9 peak region associated with baseline triglyceride levels. (PDF, 522 KB)
- Figure S3 - Liver expression of genes previously associated with markers of cardiovascular disease on Chromosome 9. (PDF, 367 KB)
- Figure S4 - Identification of a *cis*-eQTL on Chromosome 6 for *Apobec1* expression. (PDF, 362 KB)
- Table S1 - Compositions of the AIN-76A (D10001), high protein (D12083101), and atherogenic (D12109C) diets used in the study. (PDF, 114 KB)
