# Supplementary material for: High-Resolution Genetic Mapping in the Diversity Outbred Mouse Population Identifies Apobec1 as a Candidate Gene for Atherosclerosis
Source: G3 (Bethesda). 2014 Oct 23;4(12):2353–63. doi: 10.1534/g3.114.014704 (PMC4267931; doi:10.1534/g3.114.014704)
Supplement: Supporting Information [file supp_g3.114.014704_014704SI.pdf]

G3 · Genes | Genomes | Genetics

High-Resolution Genetic Mapping in the Diversity Outbred Mouse Population Identifies *Apobec1* as a Candidate Gene for Atherosclerosis

Tangi L. Smallwood[1], Daniel M. Gatti[2], Pamela Quizon[3], George M. Weinstock[4], Kuo-Chen Jung[1], Liyang Zhao[5], Kunjie Hua[1], Daniel Pomp[1,5], Brian J. Bennett[1,3,5]

1. Department of Genetics, University of North Carolina Chapel Hill, North Carolina 27599
2. The Jackson Laboratory, Bar Harbor ME 04609
3. Nutrition Research Institute, University of North Carolina Kannapolis, North Carolina 28081
4. Jackson Laboratory for Genomic Medicine, Farmington CT
5. Department of Nutrition, University of North Carolina Chapel Hill, North Carolina 27599

Corresponding Author:
Brian J. Bennett
500 Laureate Way, Suite 2303
Kannapolis, NC 28081
704-250-5044
bennettb@email.unc.edu

DOI: 10.1534/g3.114.014704

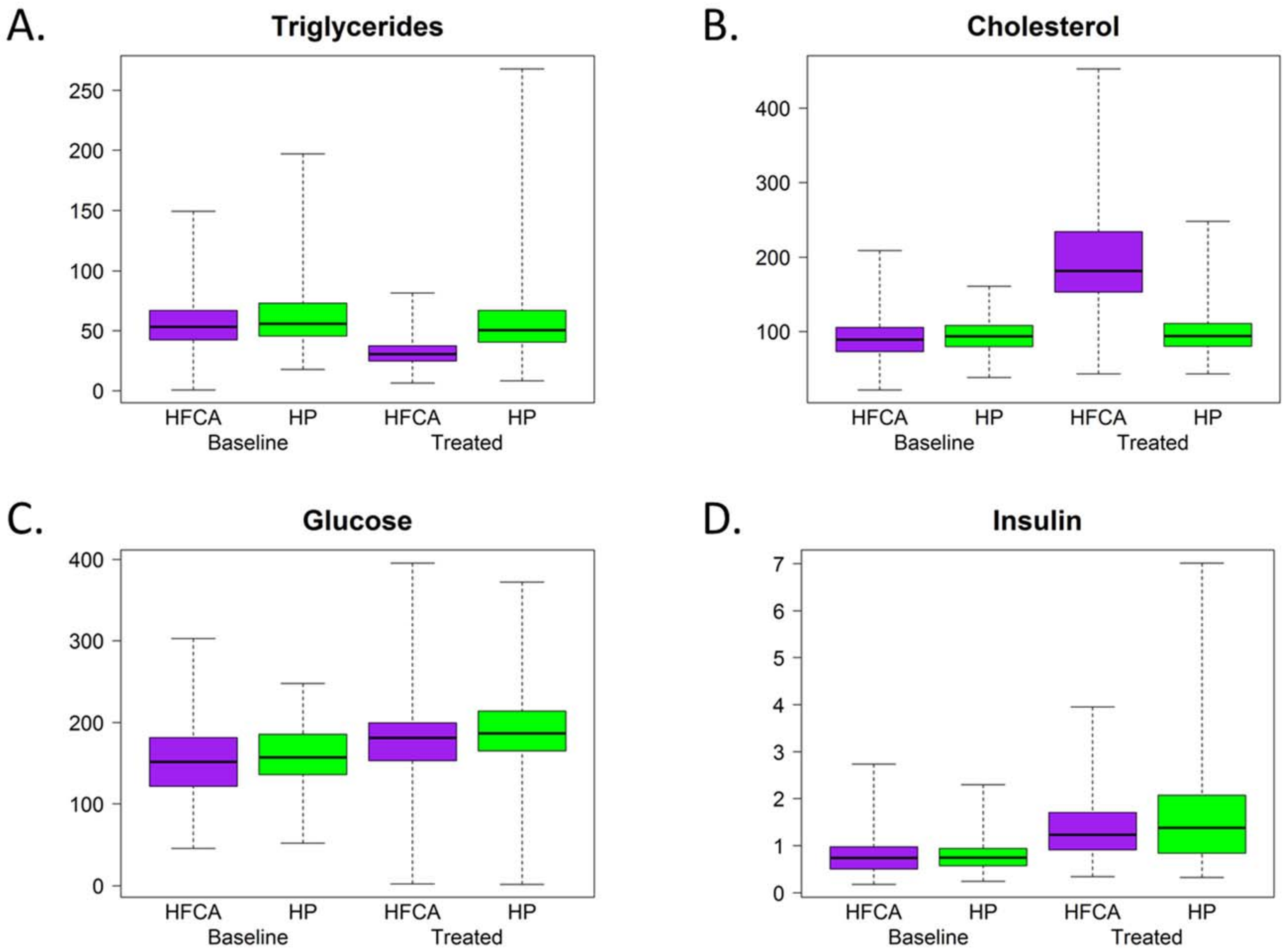

Figure S1 Effects of Diet on Cardiovascular Risk Factors in Diversity Outbred Mice. Mice were maintained on a synthetic diet for two weeks, fasted for four hours, and then phenotyped for plasma clinical chemistries at 6 weeks of age (Baseline). Following two weeks of synthetic diet, mice were transferred to either a

high protein diet (HP) or an atherogenic diet (HFCA). Plasma was taken from 24-week-old mice after 18 weeks on their respective diets, and with four hours fasting, and then phenotyped for plasma clinical chemistries after diet treatment (Treated).

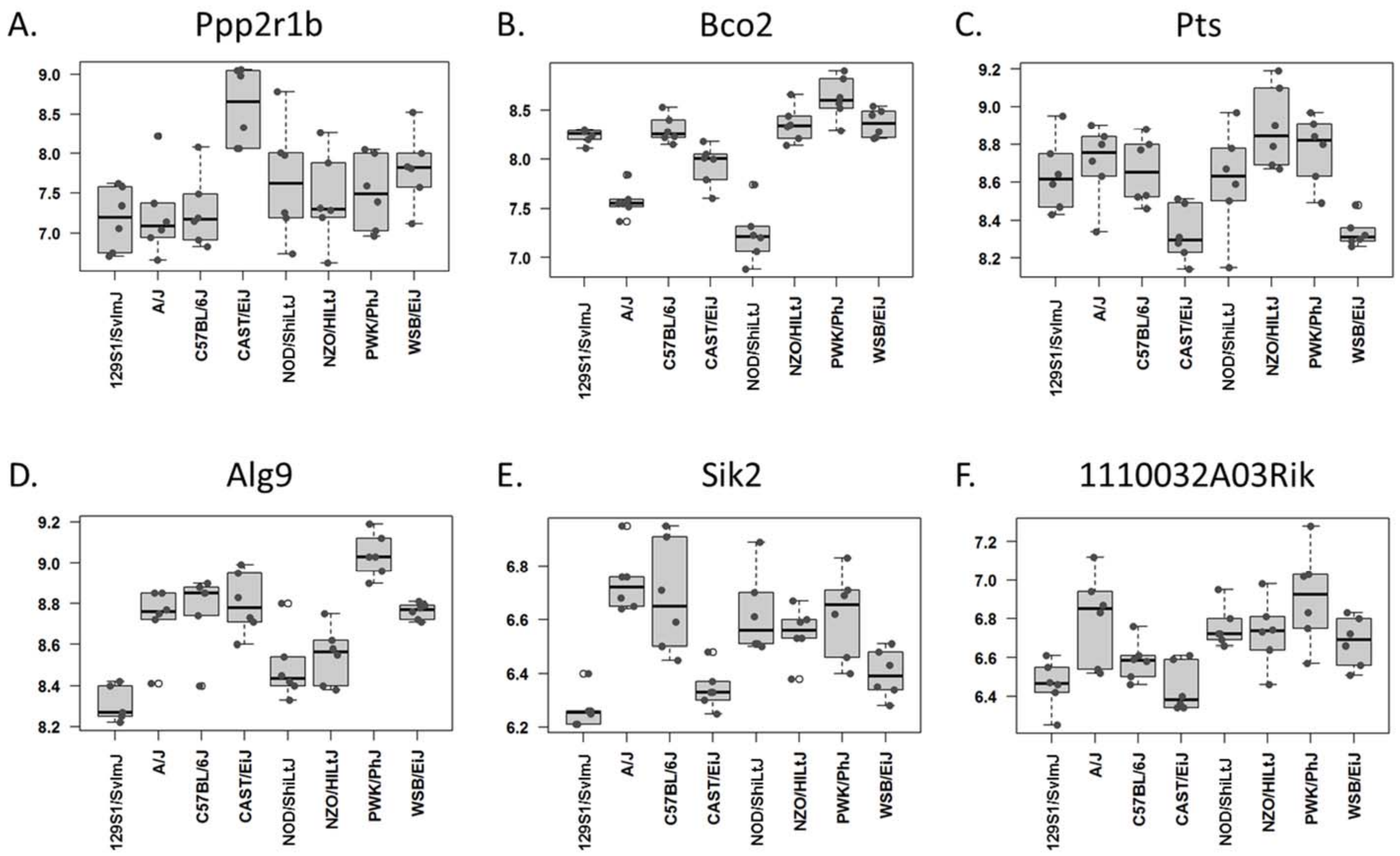
A.
Ppp2r1b
B.
Bco2
C.
Pts
D.
Alg9
E.
Sik2
F.
1110032A03Rik
129S1/SvImJ
A/J
C57BL/6J
CAST/EiJ
NOD/ShiLtJ
NZO/HlLtJ
PWK/PhJ
WSB/EiJ

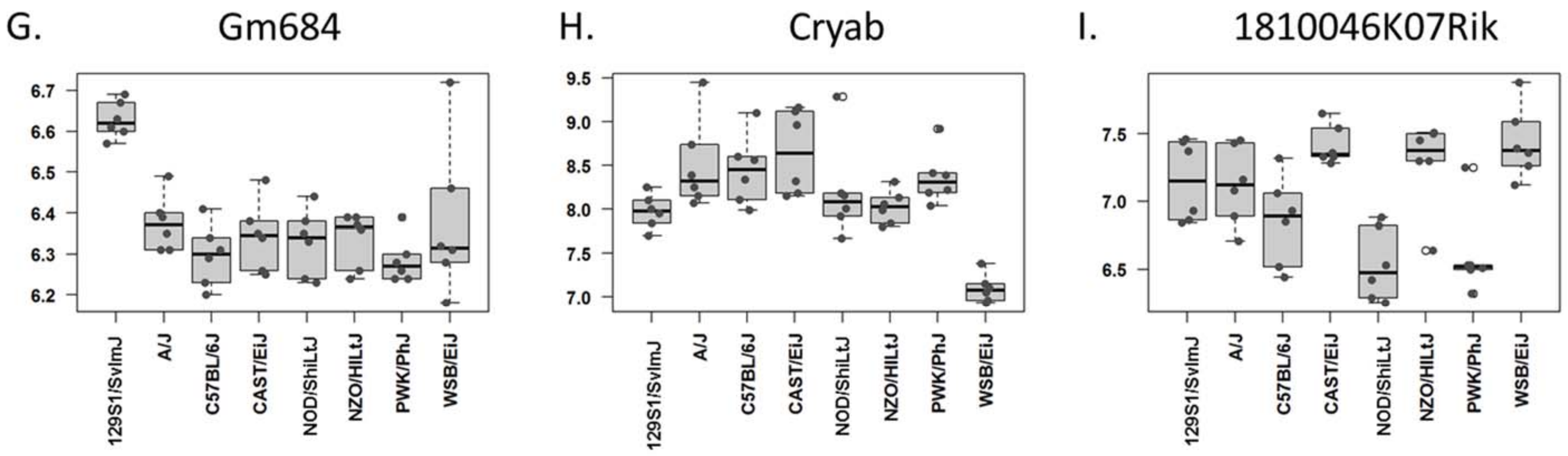

Figure S2 Liver expression of candidate genes in the Chromosome 9 peak region associated with baseline triglyceride levels. Gene expression data was obtained from livers of female C57BL6/J, A/J, NOD/ShiLtJ, NZO/HiLtJ, WSB/EiJ, CAST/EiJ, PWK/PhJ and 129S1/SvImJ mice (http://cgd.jax.org/gem/strainsurvey26). Hepatic gene expression of 68 probes representing the 34 genes at the locus were compared. Differential expression of genes across the founder strains was determined using a 1-way ANOVA analysis and a Bonferroni correction for multiple tests. We identified 9 genes as differentially expressed among the founder strains: *Ppp2r1b, Bco2, Pts, Alg9, Sik2, 1110032A03Rik, Gm684, Cryab,* and *1810046K07Rik* (A.–I.). *Bco2* and *Ppp2r1b* are differentially expressed between CAST/EiJ and the other progenitor strains and, therefore, most closely match the allele effects of the QTL.

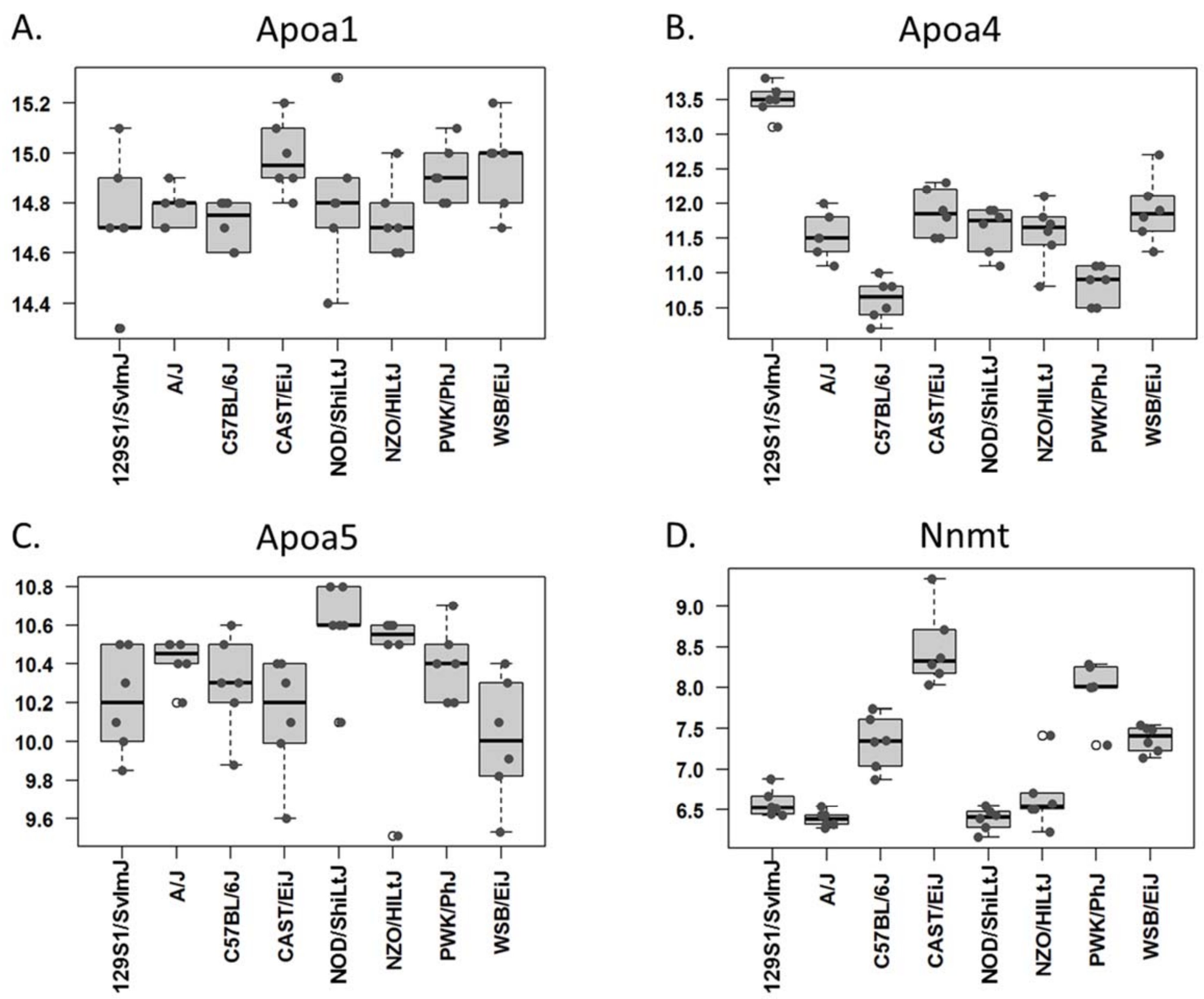

Figure S3 Liver expression of genes previously associated with markers of cardiovascular disease on Chromosome 9. Gene expression data was obtained from livers of female C57BL6/J, A/J, NOD/ShiLtJ, NZO/HiLtJ, WSB/EiJ, CAST/EiJ, PWK/PhJ and 129S1/SvImJ mice

(http://cgd.jax.org/gem/strainsurvey26). Hepatic gene expression of Chromosome 9 genes previously identified as associated with triglyceride levels were compared. Differential expression of genes across the founder strains was determined using a 1-way ANOVA analysis and a Bonferroni correction for multiple tests. *Apoa1 and Apoa5* were not differentially expressed across the founder strains (A. and C.). *Apoa4* was differentially expressed among the founder strains, but only *Nnmt* was differentially expressed in CAST/EiJ compared to all of the other founder strains (B. and D.).

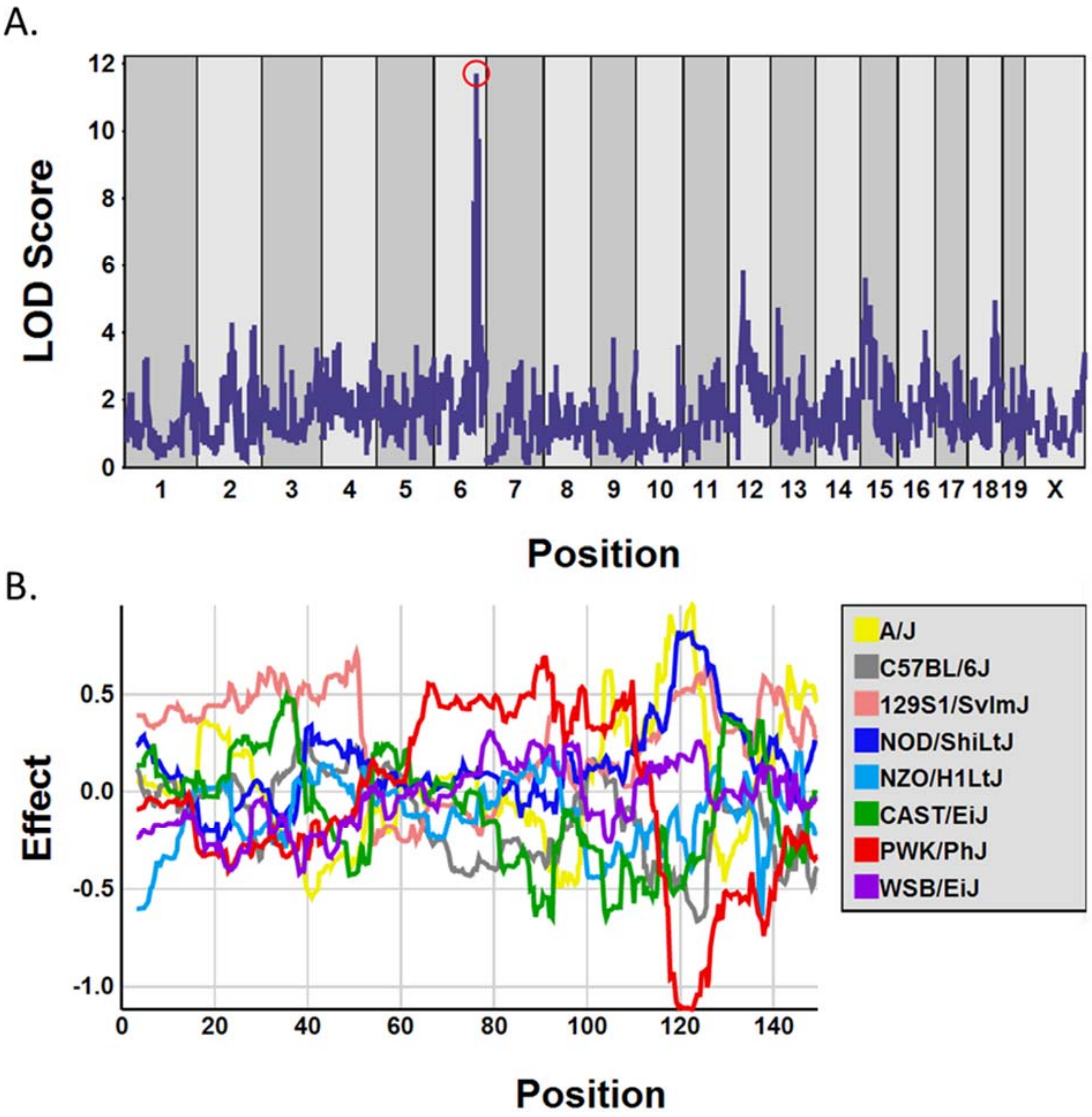

Figure S4 Identification of a *cis*-eQTL on Chromosome 6 for *Apobec1* expression. The Jackson Laboratory's Diversity Outbred eQTL viewer data located at http://cgd.jax.org/apps/eqtlviewer-beta/ was queried for eQTL associated with *Apobec1* mRNA expression. A *cis*-eQTL on Chromosome 6 was identified as

associated with *Apobec1* expression in the DO mice (LOD= 11.7). The eight coefficients of the QTL model show the effects on the phenotype contributed by each founder haplotype on Chromosome 6 (B.). These data demonstrate that A/J alleles are associated with higher expression of *Apobec1*.

Table S1 Compositions of the AIN-76A (D10001), high protein (D12083101), and atherogenic (D12109C) diets used in the study. The three diets used in this study were manufactured by Research Diets. AIN-76A was fed to the study population from 4-6 weeks of age in order to ensure that there were no spurious effects due to the potential variable composition of standard laboratory chow. Mice were then fed either D12109C or D12083101 for 18 weeks from 6-24 weeks of age. These diets differed by composition, specifically in terms of fat: protein ratio and cholesterol content, D12083101 containing 5% fat and 20.3% protein and D12109C containing 20% fat, 1.25% cholesterol, and 0.5% cholic acid. D12109C is considered atherogenic and was intended to induce the formation of atherosclerosis in the DO mice.

**Product #**	**D10001**		**D12083101**		**D12109C**	
**%**	**gm**	**kcal**	**gm**	**kcal**	**gm**	**kcal**
Protein	20.3	20.8	40.6	40	22.5	20
Carbohydrate	66.0	67.7	40.6	40	45	40
Fat	5.0	11.5	9.1	20	20	40
Total	91.3	100	90.3	100	87.5	100
kcal/gm	3.90		4.07		4.5	
**Ingredient**	**gm**	**kcal**	**gm**	**kcal**	**gm**	**kcal**
Casein, Lactic	0	0	400	1600	200	800
Casein, 30 Mesh	200	800	0	0	0	0
L-Cystine	0	0	6	24	3	12
DL-Methionine	3	12	0	0	0	0
Corn Starch	150	600	212	848	212	848
Maltodextrin 10	0	0	71	284	71	284
Sucrose	500	2000	113	452	113	452
Cellulose, BW200	50	0	50	0	50	0
Corn Oil	50	450	0	0	0	0
Soybean Oil	0	0	25	225	25	225
Cocoa Butter	0	0	66	594	155	1395
Mineral Mix S10001	35	0	0	0	0	0
Mineral Mix S10021	0	0	10	0	10	0
Dicalcium Phosphate	0	0	13	0	13	0
Calcium Carbonate	0	0	5.5	0	5.5	0
Potassium Citrate	0	0	16.5	0	16.5	0
Vitamin Mix V10001	10	40	10	40	10	40
Choline Bitartrate	2	0	2	0	2	0
Cholesterol	0	0	0	0	11.25	0
Sodium Cholate	0	0	0	0	4.5	0
Red Dye	0	0	0	0	0.05	0
Blue Dye	0	0	0.05	0	0.05	0
Yellow Dye	0	0	0.05	0	0	0
**Total**	**1000**	**3902**	**1000.1**	**4067**	**901.85**	**4056**
