# Supplementary material for: High-Resolution Genetic Mapping in the Diversity Outbred Mouse Population Identifies Apobec1 as a Candidate Gene for Atherosclerosis
Source: G3 (Bethesda). 2014 Oct 23;4(12):2353–63. doi: 10.1534/g3.114.014704 (PMC4267931; doi:10.1534/g3.114.014704)
Supplement: Supporting Information [file supp_g3.114.014704_FigureS1.pdf]

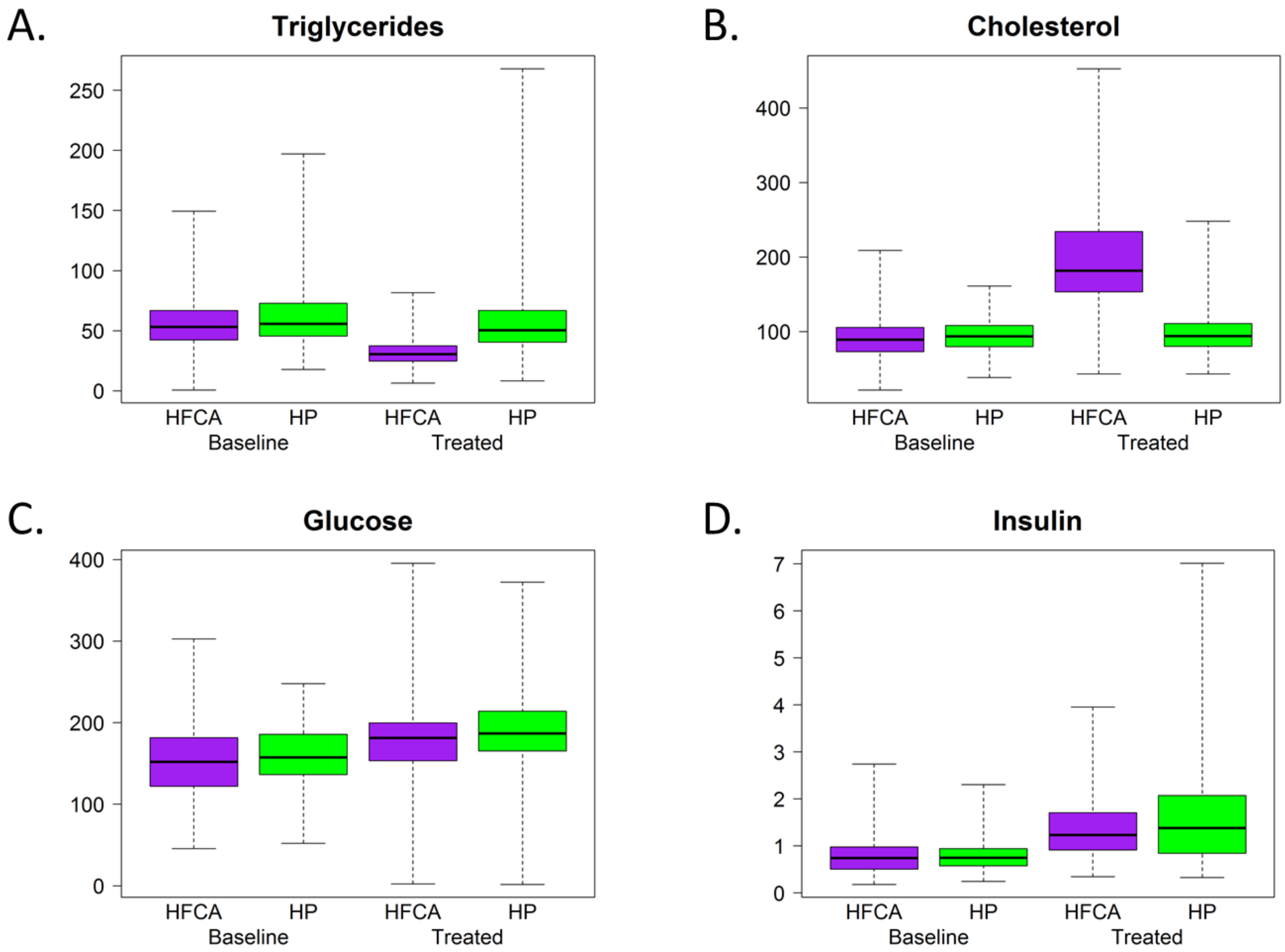

Figure S1 Effects of Diet on Cardiovascular Risk Factors in Diversity Outbred Mice. Mice were maintained on a synthetic diet for two weeks, fasted for four hours, and then phenotyped for plasma clinical chemistries at 6 weeks of age (Baseline). Following two weeks of synthetic diet, mice were transferred to either a

high protein diet (HP) or an atherogenic diet (HFCA). Plasma was taken from 24-week-old mice after 18 weeks on their respective diets, and with four hours fasting, and then phenotyped for plasma clinical chemistries after diet treatment (Treated).
