# Supplementary material for: High-Resolution Genetic Mapping in the Diversity Outbred Mouse Population Identifies Apobec1 as a Candidate Gene for Atherosclerosis
Source: G3 (Bethesda). 2014 Oct 23;4(12):2353–63. doi: 10.1534/g3.114.014704 (PMC4267931; doi:10.1534/g3.114.014704)
Supplement: Supporting Information [file supp_g3.114.014704_FigureS2.pdf]

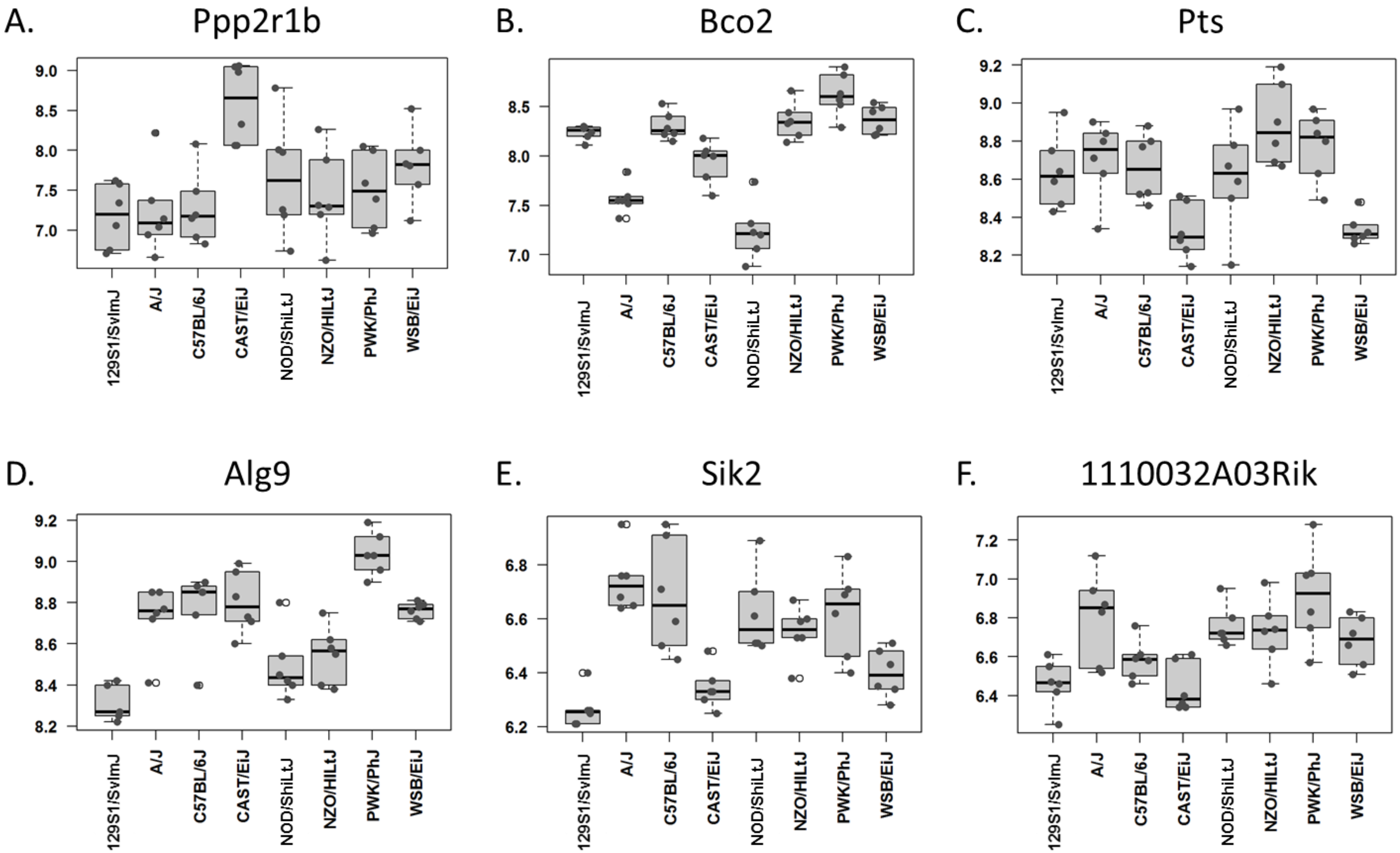
A.
Ppp2r1b
B.
Bco2
C.
Pts
D.
Alg9
E.
Sik2
F.
1110032A03Rik
129S1/SvImJ
A/J
C57BL/6J
CAST/EiJ
NOD/ShiLtJ
NZO/HlLtJ
PWK/PhJ
WSB/EiJ

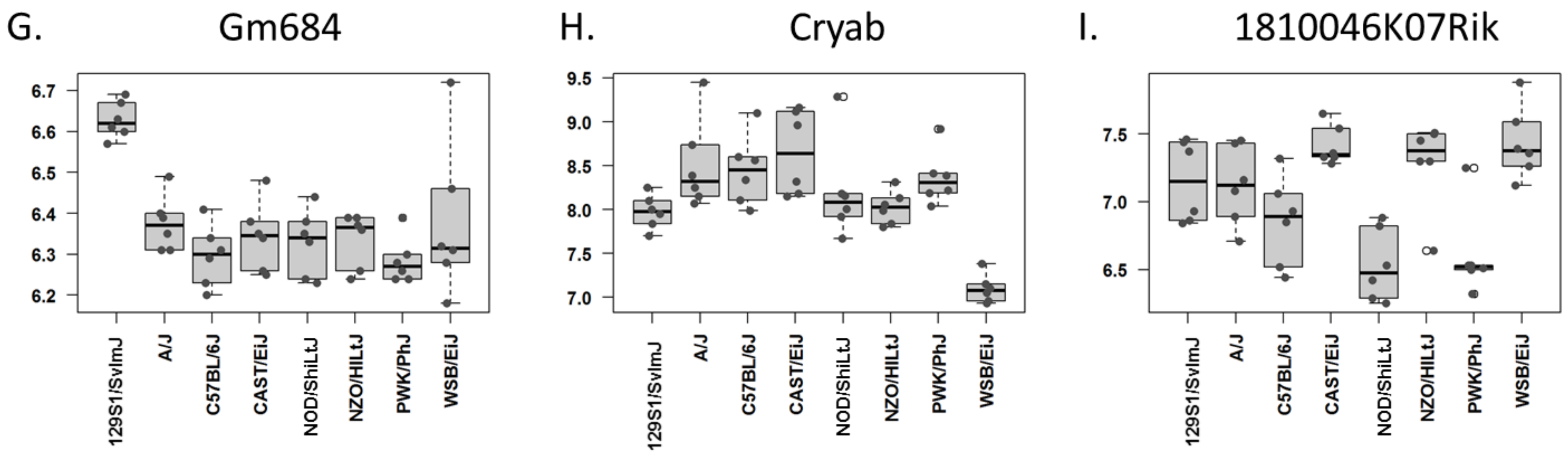

Figure S2 Liver expression of candidate genes in the Chromosome 9 peak region associated with baseline triglyceride levels. Gene expression data was obtained from livers of female C57BL6/J, A/J, NOD/ShiLtJ, NZO/HiLtJ, WSB/EiJ, CAST/EiJ, PWK/PhJ and 129S1/SvImJ mice (http://cgd.jax.org/gem/strainsurvey26). Hepatic gene expression of 68 probes representing the 34 genes at the locus were compared. Differential expression of genes across the founder strains was determined using a 1-way ANOVA analysis and a Bonferroni correction for multiple tests. We identified 9 genes as differentially expressed among the founder strains: *Ppp2r1b, Bco2, Pts, Alg9, Sik2, 1110032A03Rik, Gm684, Cryab,* and *1810046K07Rik* (A.–I.). *Bco2* and *Ppp2r1b* are differentially expressed between CAST/EiJ and the other progenitor strains and, therefore, most closely match the allele effects of the QTL.
