# Supplementary material for: High-Resolution Genetic Mapping in the Diversity Outbred Mouse Population Identifies Apobec1 as a Candidate Gene for Atherosclerosis
Source: G3 (Bethesda). 2014 Oct 23;4(12):2353–63. doi: 10.1534/g3.114.014704 (PMC4267931; doi:10.1534/g3.114.014704)
Supplement: Supporting Information [file supp_g3.114.014704_FigureS3.pdf]

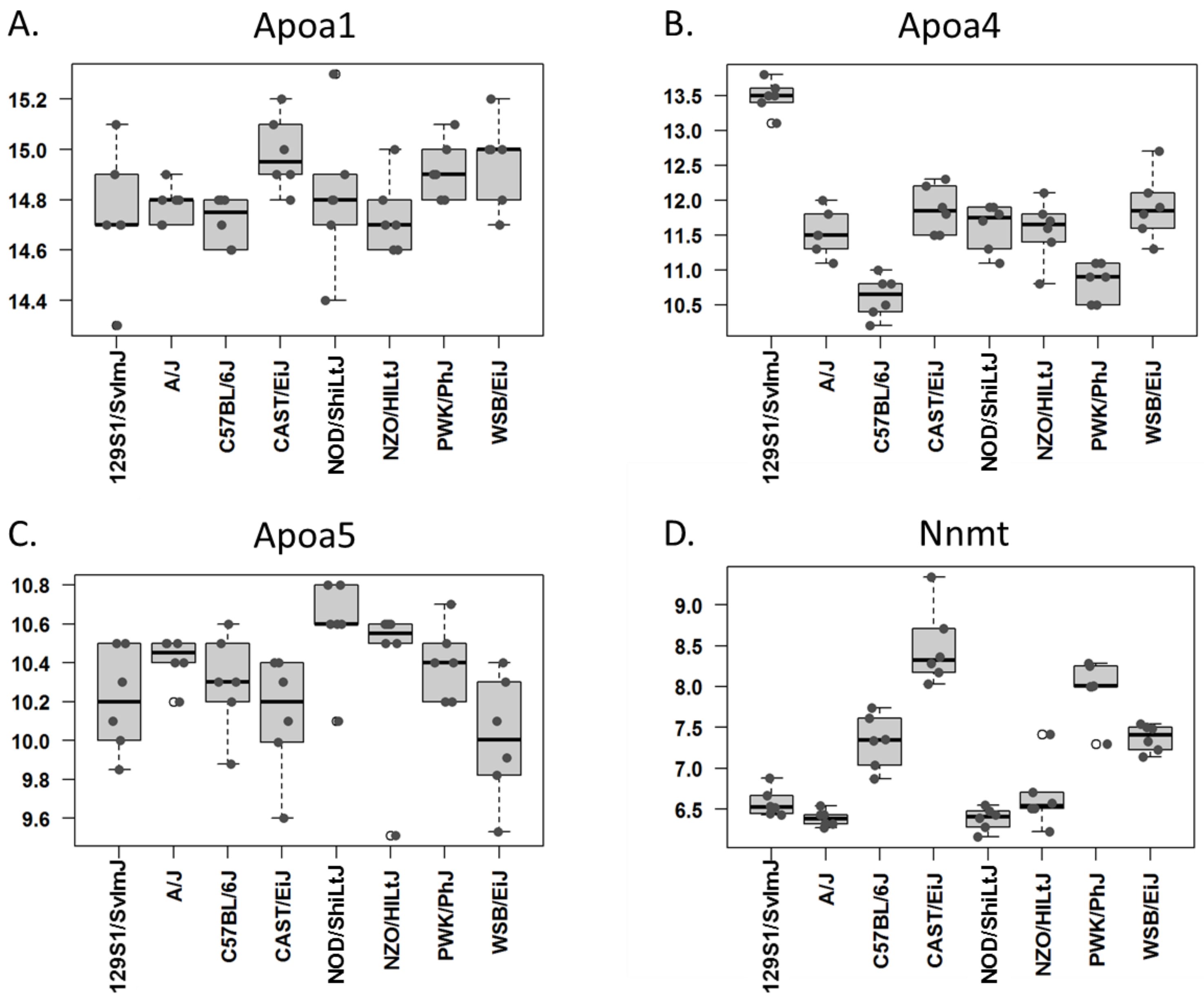

Figure S3 Liver expression of genes previously associated with markers of cardiovascular disease on Chromosome 9. Gene expression data was obtained from livers of female C57BL6/J, A/J, NOD/ShiLtJ, NZO/HiLtJ, WSB/EiJ, CAST/EiJ, PWK/PhJ and 129S1/SvImJ mice

(http://cgd.jax.org/gem/strainsurvey26). Hepatic gene expression of Chromosome 9 genes previously identified as associated with triglyceride levels were compared. Differential expression of genes across the founder strains was determined using a 1-way ANOVA analysis and a Bonferroni correction for multiple tests. *Apoa1 and Apoa5* were not differentially expressed across the founder strains (A. and C.). *Apoa4* was differentially expressed among the founder strains, but only *Nnmt* was differentially expressed in CAST/EiJ compared to all of the other founder strains (B. and D.).
