# Supplementary material for: High-Resolution Genetic Mapping in the Diversity Outbred Mouse Population Identifies Apobec1 as a Candidate Gene for Atherosclerosis
Source: G3 (Bethesda). 2014 Oct 23;4(12):2353–63. doi: 10.1534/g3.114.014704 (PMC4267931; doi:10.1534/g3.114.014704)
Supplement: Supporting Information [file supp_g3.114.014704_FigureS4.pdf]

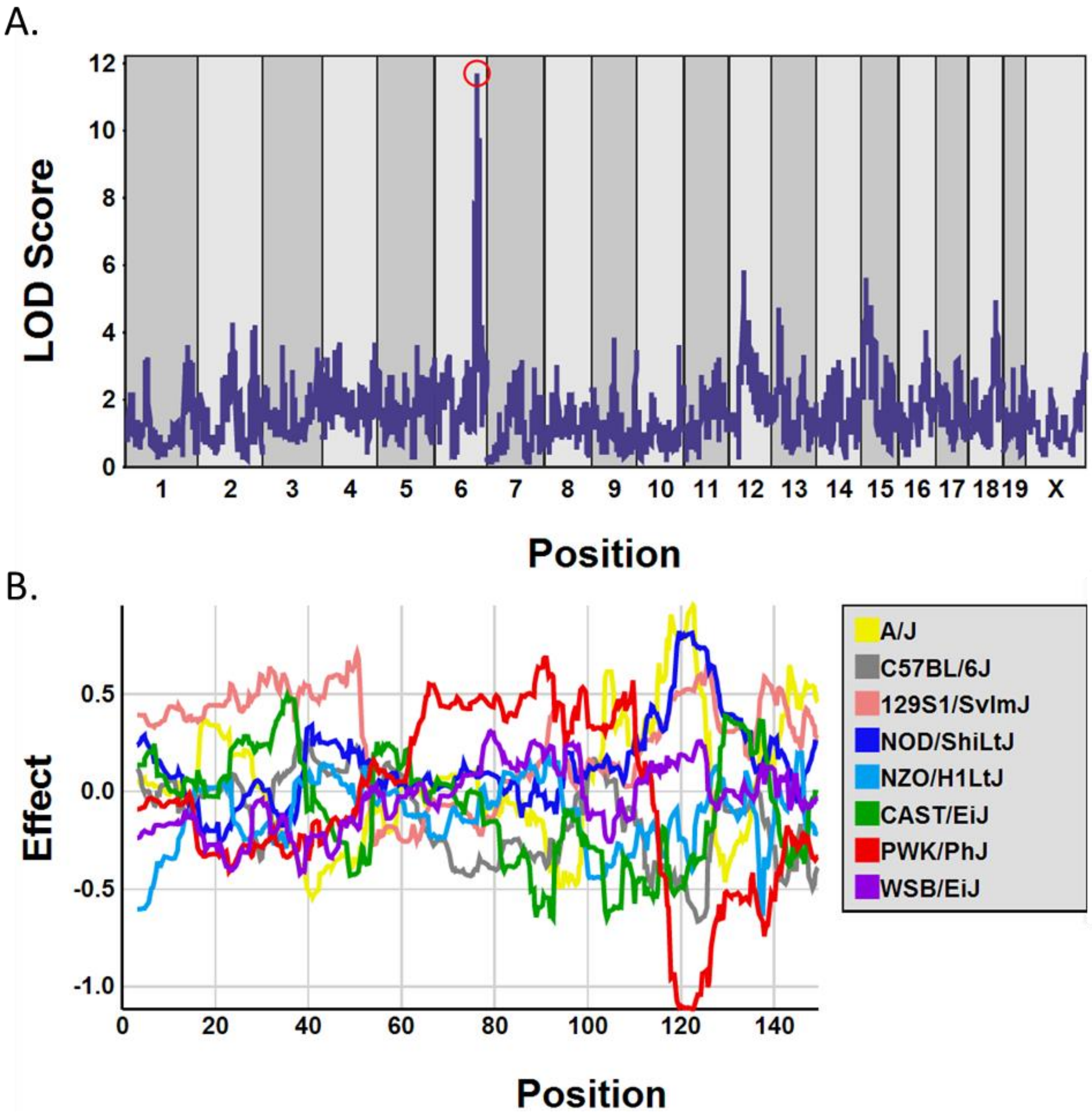

Figure S4 Identification of a *cis*-eQTL on Chromosome 6 for *Apobec1* expression. The Jackson Laboratory's Diversity Outbred eQTL viewer data located at http://cgd.jax.org/apps/eqtlviewer-beta/ was queried for eQTL associated with *Apobec1* mRNA expression. A *cis*-eQTL on Chromosome 6 was identified as

associated with *Apobec1* expression in the DO mice (LOD= 11.7). The eight coefficients of the QTL model show the effects on the phenotype contributed by each founder haplotype on Chromosome 6 (B.). These data demonstrate that A/J alleles are associated with higher expression of *Apobec1*.
