# Supplementary material for: High-Resolution Genetic Mapping in the Diversity Outbred Mouse Population Identifies Apobec1 as a Candidate Gene for Atherosclerosis
Source: G3 (Bethesda). 2014 Oct 23;4(12):2353–63. doi: 10.1534/g3.114.014704 (PMC4267931; doi:10.1534/g3.114.014704)
Supplement: Supporting Information [file supp_g3.114.014704_TableS1.pdf]

Table S1 Compositions of the AIN-76A (D10001), high protein (D12083101), and atherogenic (D12109C) diets used in the study. The three diets used in this study were manufactured by Research Diets. AIN-76A was fed to the study population from 4-6 weeks of age in order to ensure that there were no spurious effects due to the potential variable composition of standard laboratory chow. Mice were then fed either D12109C or D12083101 for 18 weeks from 6-24 weeks of age. These diets differed by composition, specifically in terms of fat: protein ratio and cholesterol content, D12083101 containing 5% fat and 20.3% protein and D12109C containing 20% fat, 1.25% cholesterol, and 0.5% cholic acid. D12109C is considered atherogenic and was intended to induce the formation of atherosclerosis in the DO mice.

**Product #**	**D10001**		**D12083101**		**D12109C**	
**%**	**gm**	**kcal**	**gm**	**kcal**	**gm**	**kcal**
Protein	20.3	20.8	40.6	40	22.5	20
Carbohydrate	66.0	67.7	40.6	40	45	40
Fat	5.0	11.5	9.1	20	20	40
Total	91.3	100	90.3	100	87.5	100
kcal/gm	3.90		4.07		4.5	
**Ingredient**	**gm**	**kcal**	**gm**	**kcal**	**gm**	**kcal**
Casein, Lactic	0	0	400	1600	200	800
Casein, 30 Mesh	200	800	0	0	0	0
L-Cystine	0	0	6	24	3	12
DL-Methionine	3	12	0	0	0	0
Corn Starch	150	600	212	848	212	848
Maltodextrin 10	0	0	71	284	71	284
Sucrose	500	2000	113	452	113	452
Cellulose, BW200	50	0	50	0	50	0
Corn Oil	50	450	0	0	0	0
Soybean Oil	0	0	25	225	25	225
Cocoa Butter	0	0	66	594	155	1395
Mineral Mix S10001	35	0	0	0	0	0
Mineral Mix S10021	0	0	10	0	10	0
Dicalcium Phosphate	0	0	13	0	13	0
Calcium Carbonate	0	0	5.5	0	5.5	0
Potassium Citrate	0	0	16.5	0	16.5	0
Vitamin Mix V10001	10	40	10	40	10	40
Choline Bitartrate	2	0	2	0	2	0
Cholesterol	0	0	0	0	11.25	0
Sodium Cholate	0	0	0	0	4.5	0
Red Dye	0	0	0	0	0.05	0
Blue Dye	0	0	0.05	0	0.05	0
Yellow Dye	0	0	0.05	0	0	0
**Total**	**1000**	**3902**	**1000.1**	**4067**	**901.85**	**4056**
